# Interlayer-active layered oxysulfides NaMTiO_2.2_S_1.8_ (M = Nd, Sm) with an *n* = 1 Ruddlesden–Popper structure acting as photocatalysts for visible light water splitting

**DOI:** 10.1039/d5sc04851f

**Published:** 2025-08-13

**Authors:** Yusuke Ishii, Hajime Suzuki, Daichi Kato, Osamu Tomita, Akinobu Nakada, Ryu Abe

**Affiliations:** a Department of Energy and Hydrocarbon Chemistry, Graduate School of Engineering, Kyoto University Katsura, Nishikyo-ku Kyoto 615-8510 Japan

## Abstract

Layered compounds that utilize interlayer space as a reactive field are known as “interlayer-active” compounds and have been gaining attention, particularly in photocatalysis for water splitting. However, most of the reported “interlayer-active” photocatalysts are oxide semiconductors that possess a wide bandgap. Thus, they cannot utilize visible light essential for efficient water splitting. In this study, we synthesized novel Ruddlesden–Popper (RP) (*n* = 1) layered oxysulfides, NaMTiO_2.2_S_1.8_ (M = Nd, Sm), by heating “interlayer-active” layered oxides, NaMTiO_4_, under H_2_S flow. In NaMTiO_2.2_S_1.8_, the sulfur atoms occupy the apical oxygen sites and contribute to the elevated valence band maximum (VBM) to enable visible light absorption. Additionally, NaMTiO_2.2_S_1.8_ exhibits both proton exchange and interlayer hydration capabilities as well as photocatalytic activity for hydrogen and oxygen evolution under visible light. Hence, NaMTiO_2.2_S_1.8_ is the first example of both a *n* = 1 RP and an “interlayer-active” oxysulfide with the potential for visible-light-driven overall water splitting. The “interlayer-active” RP (*n* = 1) oxysulfide is expected to find application in various fields beyond photocatalysis by utilizing interlayer reactions such as ion exchange and interlayer hydration.

## Abstract 2

We report novel Ruddlesden–Popper (*n* = 1) oxysulfides, NaMTiO_2.2_S_1.8_ (M = Nd, Sm), the first oxysulfides exhibiting “interlayer-active” behavior, featuring ion exchange, interlayer hydration, and potential for overall photocatalytic water splitting.

## Graphical abstract

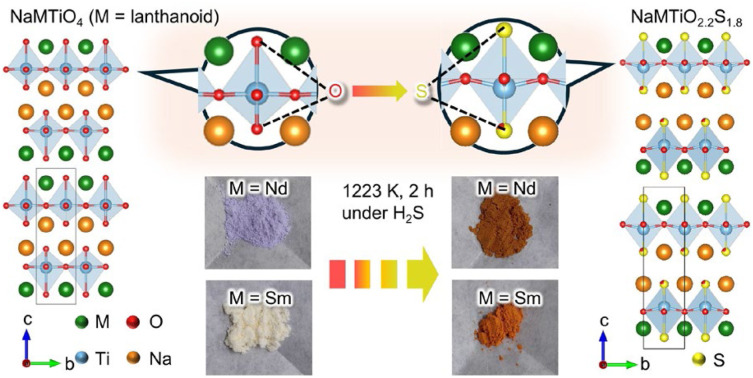

## Introduction

Layered oxides with functional interlayers have attracted considerable interest in fields such as energy storage,^1–3^ nanoelectronics,^4–6^ and photocatalysis.^7–9^ For instance, layered lithium cobalt oxide (LiCoO_2_) is a widely used cathode material in Li-ion batteries because of its strong electromotive force and excellent charge–discharge characteristics due to its outstanding lithium-ion diffusion in the interlayers.^1,10,11^ Additionally, layered NaCoO_2_ ^12,13^ and NaFeO_2_,^14,15^ which contain Na ions in the interlayer, are the most promising materials for next-generation Na-ion batteries. In nanoelectronics, various nanosheets synthesized by exfoliating layered oxides have attracted significant attention owing to their high crystallinity, derived from the parent layered oxides, as well as their high designability and rich variety.^4,16,17^ For example, Ca_2_Nb_3_O_10_ nanosheets, which are obtained by the exfoliation of layered HCa_2_Nb_3_O_10_ by exchanging the H^+^ ions in its interlayer for bulky tetrabutylammonium ions (TBA^+^), reportedly function efficiently as a gate insulator.^18^ The low leakage current density and high dielectric constant of a dielectric thin film based on Ti_0.87_O_2_ nanosheets, derived from layered H_1.07_Ti_1.73_O_4_, make it suitable for application as a field-effect transistor.^6^ In the field of photocatalysis, it has been demonstrated that certain layered metal oxides, such as K_4_Nb_6_O_17_ ^19^ and K_2_La_2_Ti_3_O_10_,^9^ which utilize their interlayer spaces as reaction sites for water splitting, are highly efficient photocatalysts for the overall water splitting reaction under UV light irradiation. The particles of common non-layered photocatalysts are several hundred nanometers in size, and photoexcited carriers must travel long distances to react with water molecules; in contrast, photoexcited carriers in layered oxides only travel within the 1–2 nm-thick metal oxide layer to react with water molecules in the interlayer. This enables the efficient utilization of photoexcited carriers in the reaction before recombination, with high quantum yields for water splitting under UV light.^20^

More recently, research interest has extended to sulfides and oxysulfides.^21–26^ Owing to the lower electronegativity (and higher polarizability) of sulfur compared to that of oxygen, sulfides tend to form anionic frameworks with greater polarizability compared to oxides. This enhanced polarizability increases the ion conductivity and makes sulfides particularly attractive for use in metal-ion batteries.^22^ Moreover, many (oxy)sulfide semiconductors can absorb visible and near-infrared light because of the low electronegativity of sulfur. Consequently, these materials are actively applied in the fields of nanoelectronics and photocatalysis, *e.g.*, in phototransistors^27,28^ and photodetectors^24,29^ sensitive to visible and near-infrared light, as well as in water-splitting photocatalysts for the generation of hydrogen from water under visible light.^25^

The valence bands of (oxy)sulfide semiconductors are primarily composed of S-3p orbitals, which elevate the valence band maximum (VBM) to decrease the bandgaps compared to those of oxide semiconductors. In photocatalytic applications, many (oxy)sulfides have been developed as highly active hydrogen evolution photocatalysts upon irradiation with visible light.^25,30,31^ In particular, oxysulfide photocatalysts demonstrate higher stability against self-oxidation than sulfide photocatalysts, likely because of the partial contribution of O-2p orbitals to the valence band, which enables many of them to demonstrate activity for both water oxidation and reduction. In other words, the photogenerated holes are stably utilized for water oxidation rather than for oxidizing the sulfide anions (S^2−^) in oxysulfides (*e.g.*, S^2−^ + 2h^+^ → S^0^). Indeed, M_2_Ti_2_O_5_S_2_ (M = Y, Sm, *etc.*) reportedly stably oxidizes water to oxygen under visible light.^25,32,33^ However, none of these oxysulfides possess “active interlayer spaces” that can function as reaction sites for water splitting. In fact, layered oxysulfides with both ion exchange and interlayer hydration capabilities, so-called “interlayer-active” oxysulfides, have not been reported to date.

**Fig. 1:**
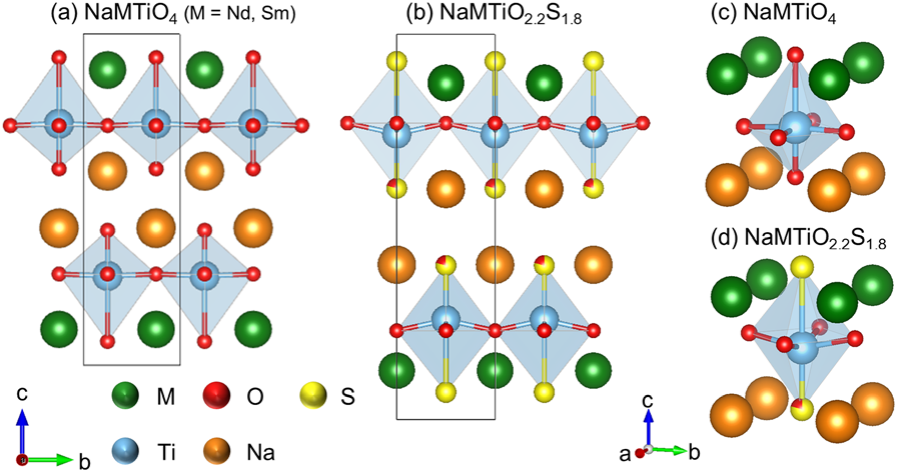
Crystal structures of (a) NaMTiO_4_ (M = Nd, Sm) and (b) NaMTiO_2.2_S_1.8_. (c and d) Enlarged views around the Ti–O(S) octahedra.

In this study, we successfully synthesized Ruddlesden–Popper (RP) (*n* = 1) layered oxysulfides, NaMTiO_2.2_S_1.8_ (M = Nd, Sm) (Fig. 1), by sulfurizing the “interlayer-active” layered oxide NaMTiO_4_ as a precursor under H_2_S flow. These compounds are the first examples of “interlayer-active” oxysulfides, and we demonstrated that they also function as photocatalysts for hydrogen/oxygen evolution under visible light. These “interlayer-active” RP (*n* = 1) oxysulfides are expected to find application beyond photocatalysis through ion-exchange reactions and interlayer hydration.

## Results and discussion

### Synthesis of new oxysulfides NaMTiO_2.2_S_1.8_*via* sulfurization of NaMTiO_4_

**Fig. 2:**
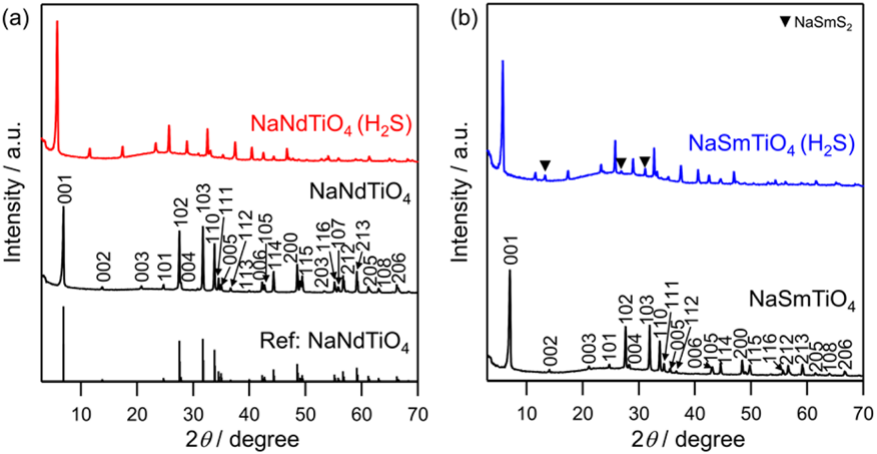
XRD patterns of NaMTiO_4_ (M = (a) Nd, (b) Sm) and the sulfurized product of the NaMTiO_4_ (NaMTiO_4_ (H_2_S)), along with reference patterns of NaNdTiO_4_ (ICSD #82004). Triangles denote the peaks of the byproduct NaSmS_2_ (ICSD #644974).

RP NaMTiO_4_ (M = Nd, Sm) was synthesized *via* a conventional solid-state reaction to serve as a precursor for sulfurization, as previously reported.^34^ X-ray diffraction (XRD) analysis confirmed the presence of single-phase NaMTiO_4_ (Fig. 2), which was then heated at 1223 K for 2 h under H_2_S flow (denoted as NaMTiO_4_ (H_2_S)). After sulfurization, the XRD pattern resembled that of the parent phase and could be indexed using the ideal tetragonal lattice as NaLaTiO_4_ (*P*4/*nmm*), except for small byproduct peaks, such as those of NaSmS_2_. However, the *c*-axis was extended significantly (Δ*c* ≈ 2.4 Å), whereas the *a*-axis did not change much (NaNdTiO_4_: *a* ≈ 3.8 Å, *c* ≈ 12.8 Å and NaNdTiO_4_ (H_2_S): *a* ≈ 3.9 Å, *c* ≈ 15.2 Å) (Fig. S1), strongly suggesting the successful substitution of O by the larger S atom at the apical site of the perovskite layer. A substantial amount of S was confirmed by energy dispersive X-ray spectroscopy (EDX) analysis (M = Nd : S/Ti = 1.73, M = Sm : S/Ti = 1.68) (Fig. S2, S3 and Table S1). Additionally, SEM images of NaMTiO_4_ before and after sulfurization show no significant change in particle shape; the particles maintained a plate-like morphology (Fig. S4). These results support the formation of the new layered oxysulfide.

**Fig. 3:**
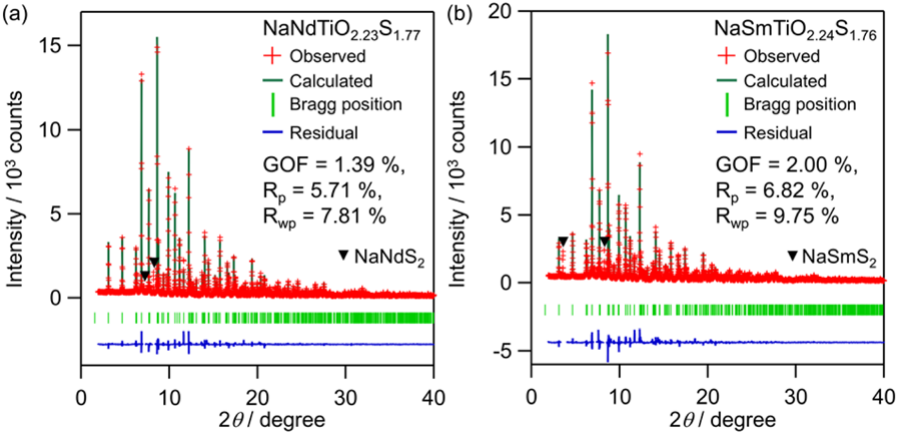
Rietveld refinement against the SXRPD patterns of (a) NaNdTiO_4_ (H_2_S) and (b) NaSmTiO_4_ (H_2_S) using structural models of NaNdTiO_2.23_S_1.77_ (Fig. S5a) and NaSmTiO_2.24_S_1.76_ (Fig. S5b). The red crosses and the green and blue lines represent the observed, calculated, and difference intensities, respectively. Green ticks indicate calculated Bragg reflections. Triangles denote the peaks of the byproducts NaNdS_2_ (ICSD #644913) and NaSmS_2_.

**Fig. 4:**
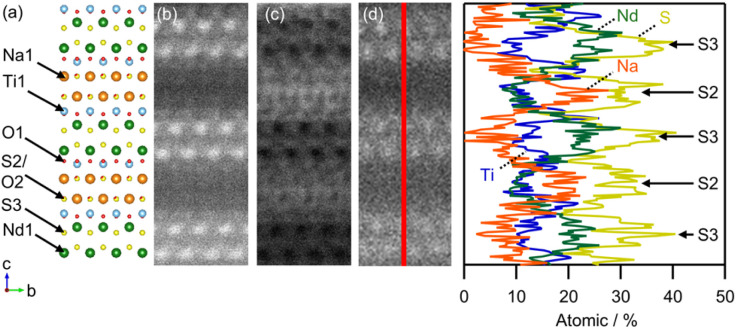
STEM images of NaNdTiO_2.2_S_1.8_ in the [100] direction. (a) The crystal structure of NaNdTiO_2.2_S_1.8_. Enlargements of (b) HAADF and (c) ABF. (d) STEM-EDX line analysis of NaNdTiO_2.2_S_1.8_.

Fig. 3 shows the Rietveld refinement of the synchrotron X-ray powder diffraction (SXRPD) data. The structural models we constructed for the Rietveld refinement based on the NaLaTiO_4_ structure (space group *P*4/*nmm*) (Fig. S5a and b) are not structurally distorted because the SXRPD pattern does not contain any superlattice peaks. In addition, the difference in the lattice parameters before and after sulfurization is similar to that of the *n* = 2 RP oxide (*e.g.*, Sr_3_Ti_2_O_7_)^35^ and oxysulfide (*e.g.*, Y_2_Ti_2_O_5_S_2_),^36^ where selective substitution of S at the apical site of the perovskite layer elongates the *c*-axis but does not affect the *a*-axis. Density functional theory (DFT) calculation, performed using the NaLaTiO_4−*x*_S_*x*_ structure without *f*-electrons to simplify the calculation, also supports the site selectivity of sulfur at the apical site in NaMTiO_4−*x*_S_*x*_ (see discussion in Fig. S6, S7 and Tables S2, S3). Therefore, we partially replaced the apical oxygen of the ideal NaLaTiO_4_ structure with sulfur, refined all variable positional parameters, *U*_iso_, and refined the occupancy of sulfur at the apical site. The occupancy of the S3 site adjacent to M converged to 1.11(2); hence, we fixed it at unity. On the other hand, the occupancy of S at the S2 site adjacent to the Na site converged to 80%, giving the composition of NaNdTiO_2.2_S_1.8_, which is consistent with the EDX result. The *U*_iso_ of Na1 converged to a large value of 0.04 Å^2^. This large *U*_iso_ may indicate high mobility of Na in the sulfide layer due to weak Na–S interaction, as seen in sulfide-based alkali-ion conductors,^37–39^ such as Na_3_PS_4_, which could be beneficial for ion-exchange reactions. The refinement yields a good fit with the reliability factors (M = Nd: *R*_wp_ = 7.81% and GOF = 1.39; M = Sm: *R*_wp_ = 9.75% and GOF = 2.00). The final crystallographic data (Tables S4 and S5) and profile after refinement (Fig. 3) correspond with the high-angle annular dark field scanning transmission electron microscopy (HAADF-STEM) and annular bright-field STEM (ABF-STEM) images along the [100] direction (Fig. 4a–c and S8). Additionally, the STEM-EDX line analysis supported selective occupation of sulfur at the apical sites of the perovskite layer (S2 and S3) (Fig. 4d and S9).

The structures of the *n* = 1 RP oxysulfides NaMTiO_2.2_S_1.8_ (Fig. S5a and b) were compared with that of M_2_Ti_2_O_5_S_2_, which has a cation-deficient *n* = 2 RP structure, as other *n* = 1 RP oxysulfides have not yet been reported. Most of the Ti–O and Ti–S bond lengths are similar to those of M_2_Ti_2_O_5_S_2_ (Fig. S5c and d), confirming that the resolved structure is reasonable. Both apical sites of the Ti octahedra in NaMTiO_2.2_S_1.8_ were occupied by sulfur, whereas only one of them in M_2_Ti_2_O_5_S_2_ was occupied by S, probably because the anion sites bridging the two octahedra were too small for sulfur to occupy. Notably, in NaMTiO_2.2_S_1.8_, approximately 20% of the oxygen occupies the apical anion site on the Na layer side, resulting in the Ti–O2/S2 bond length being shorter than the typical Ti–O bond but longer than the typical Ti–S bond. Such a random occupation of O and S at the same site is surprising given the Hume–Rothery rule, which states that anions with significantly different ionic radii, such as O^2−^ (1.4 Å) and S^2−^ (1.84 Å), generally cannot form solid solutions. The unusual occupation of O/S may be facilitated by the flexibility of the layered structure with weak interlayer interactions, such as that in BiOX (X = Cl, Br, I), which forms a solid solution between BiOCl and BiOI despite their distinctly different ionic radii (Cl: 1.81 Å, I: 2.2 Å).^40^

**Fig. 5:**
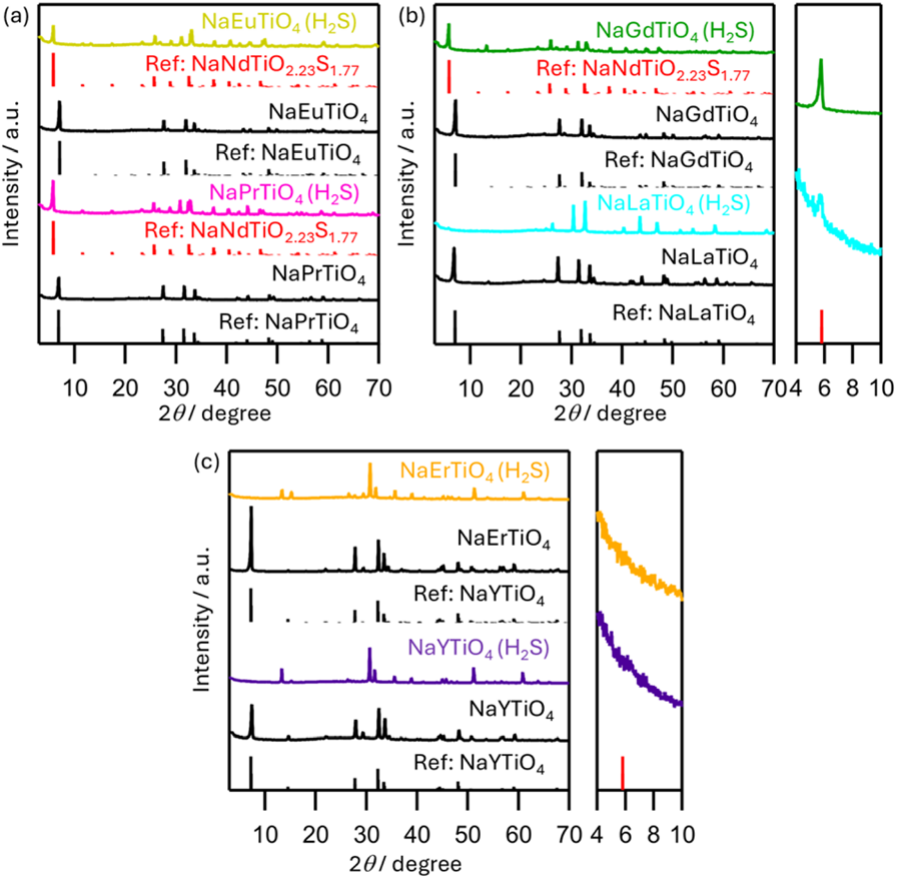
XRD patterns of NaMTiO_4_ (M = (a) Pr, Eu, (b) La, Gd, (c) Y, Er) and the sulfurized product of the NaMTiO_4_ (NaMTiO_4_ (H_2_S)), along with the reference patterns of NaPrTiO_4_ (ICSD #422048), NaEuTiO_4_ (ICSD #79229), NaGdTiO_4_ (ICSD #81537), NaLaTiO_4_ (ICSD #422047), NaNdTiO_2.23_S_1.77_ (this work), and NaYTiO_4_ (ICSD #81538).

NaMTiO_4_ (M = La, Pr, Eu, Gd, Y, and Er) were also sulfurized using the same procedure as that for Nd and Sm (Fig. 5 and S10, S11). For those metals with intermediate ionic radii, NaMTiO_4−*x*_S_*x*_ (M = Pr, Eu, Gd) was obtained as the main phase, and its lattice parameters decreased nearly linearly with the decreasing ionic radius of M (Fig. S12), indicating the successful synthesis of a series of NaMTiO_4−*x*_S_*x*_. For La with the largest ionic radius, we observed a new peak at approximately 6°, which may correspond to NaLaTiO_4−*x*_S_*x*_; however, most peaks correspond to the perovskites Na_0.5_La_0.5_TiO_3_ and NaLaS_2_ (Fig. 5b and S11a). For the smaller M cations (Y and Er), sulfurization resulted in complete decomposition into the pyrochlore phases, M_2_Ti_2_O_7_ and NaMS_2_ (Fig. 5c and S11b, c). These observations indicate that the stability of competing oxides (perovskite and pyrochlore) is important for the successful formation of the oxysulfide NaMTiO_4−*x*_S_*x*_ (Table S6).

Although RP oxides are a large family of compounds, the RP oxysulfides reported thus far are very limited, with only a few examples of *n* = 2 cation-deficient M_2_Ti_2_O_5_S_2_ and M_2_(MR)O_5_S_2_ (M = lanthanoid, Y; R = Nb and Ta). NaMTiO_4−*x*_S_*x*_ is the first reported oxysulfide with an *n* = 1 perovskite layer and A-site (Na/M) cation ordering, suggesting the possibility of expanding the oxysulfide RP family. The most notable feature of our new compounds is the presence of a rock salt layer composed of mobile Na^+^ ions. For oxide RP phases, the alkali metal layer allows ion exchange with various elements and molecules, as well as interlayer hydration, which confers unique functionalities. For example, replacing Na^+^ in NaMTiO_4_ (M = La, Nd) with H^+^, followed by substitution with a bulky cation such as TBA^+^, allows the fabrication of oxide nanosheets.^41^ Furthermore, ion-exchange reactions between alkali metals and transition metals can produce unique magnetic materials such as ferromagnetic Li_0.3_Ni_0.85_La_2_Ti_3_O_10_.^42^ In addition to ion exchange, interlayer hydration can enhance the two-dimensional nature by expanding the interlayer distance with the emergence of interesting properties, such as the superconductivity of Na_*x*_CoO_2_·*y*H_2_O induced by H_2_O intercalation.^43^ This suggests that similar ion exchange, exfoliation, and interlayer hydration chemistry could be expected for NaMTiO_2.2_S_1.8_, which potentially paves a new direction for the functional use of RP oxysulfides.

**Fig. 6:**
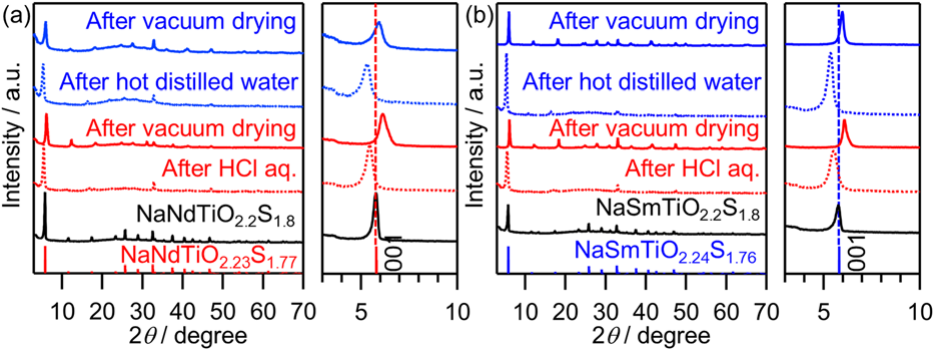
XRD patterns of the sulfurized products, NaMTiO_2.2_S_1.8_ (M = (a) Nd, (b) Sm) and the samples after stirring under dark conditions in an HCl aqueous solution for 24 h or hot distilled water for 72 h, followed by vacuum drying, along with the reference patterns of NaNdTiO_2.23_S_1.77_ (this work) and NaSmTiO_2.24_S_1.76_ (this work).

Herein, we experimentally confirmed the proton exchange and/or hydration capabilities in the interlayer of NaMTiO_2.2_S_1.8_ (M = Nd, Sm). After stirring in an aqueous HCl, the XRD patterns showed an evident shift of the (001) peak toward a lower angle (Fig. 6), indicating an increase in the interlayer distance. After HCl treatment, the lattice parameters became *a* = 4.142(8) Å and *c* = 16.31(4) Å for M = Nd and *a* = 4.136(9) Å and *c* = 16.26(3) Å for M = Sm (Fig. S13a and Table S7). The elongation of the *c*-axis (Δ*c* ≈ 1.0 Å) is comparable to the hydration of NaMTiO_4_,^44,45^ indicating the intercalation of water molecules between the layers, along with partial H^+^ exchange (*e.g.*, in the form of H_3_O^+^). EDX analysis confirmed the proton exchange: the Na/Ti molar ratio decreased from 1.12 (Nd) and 1.06 (Sm) to 0.18 (Nd) and 0.11 (Sm) after stirring in an HCl solution (Table S8). In addition, vacuum drying shifted the peak toward a higher angle owing to dehydration. For the dehydrated samples, the peak appears at a higher angle compared to the (001) peak of the original NaMTiO_2.2_S_1.8_, indicating a smaller *c*-axis lattice parameter (M = Nd: Δ*c* ≈ 0.8 Å, M = Sm: Δ*c* ≈ 0.7 Å) (Fig. S13b and Table S7). This difference also indicated the exchange of Na^+^ in NaMTiO_2.2_S_1.8_ for H^+^ with its smaller ionic radius. H^+^ exchange and hydration were also observed after stirring in distilled water, although the amount of residual Na is larger (0.27 for Nd and 0.18 for Sm) (Fig. 6 and Table S8). Additionally, in distilled water, NaMTiO_2.2_S_1.8_, especially M = Sm, required a higher reaction temperature (Fig. S14). Based on the above findings, it has been confirmed that NaMTiO_2.2_S_1.8_ is an “interlayer-active” oxysulfide, possessing both proton exchange and interlayer hydration capabilities.

### Optical properties and band structures of NaMTiO_2.2_S_1.8_ (M = Nd, Sm)

**Fig. 7:**
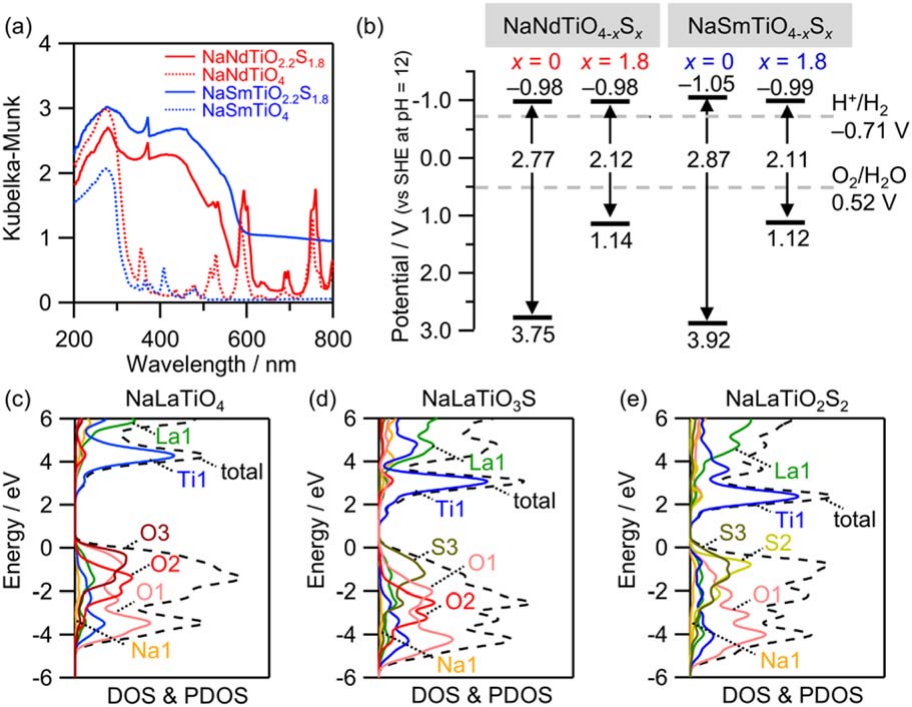
(a) UV-vis diffuse reflectance spectra and (b) band edge positions at pH = 12 of NaMTiO_4_ and NaMTiO_2.2_S_1.8_ (M = Nd, Sm). DOS and PDOS near the VBM and CBM of (c) NaLaTiO_4_, (d) NaLaTiO_3_S, and (e) NaLaTiO_2_S_2_ calculated using the structural models shown in Fig. S18.

The light absorption properties of NaMTiO_2.2_S_1.8_ (NaMTiO_4_ (H_2_S)) (M = Nd and Sm) differed from those of the oxide precursor, NaMTiO_4_, after sulfurization, and the absorption edge shifted from approximately 340 nm to approximately 600 nm (Fig. 7a). Likewise, while the presence of impurities in the Pr, Eu, and Gd samples, these absorption edges shifted toward longer wavelengths (Fig. S15). The band levels of NaMTiO_2.2_S_1.8_ (M = Nd, Sm) and NaMTiO_4_ (Fig. 7b) were estimated based on a combination of the band gaps and flat-band potentials determined from the reflectance spectra and MS plots (Fig. S16), respectively. The flat-band potentials were assumed to be located just below the conduction band maxima (CBM) owing to the n-type nature of these materials. Although the CBMs of the oxysulfides were located at potentials similar to those of the precursor oxides, their VBMs shifted significantly toward more negative potentials, rationalizing the significant decrease in the bandgap after sulfurization. The negative shift in the VBMs was also confirmed by the ionization energies obtained using photoelectron yield spectroscopy (PYS) (Fig. S17). Importantly, these new oxysulfides possess appropriate band levels for both the reduction and oxidation of water under visible light irradiation.

The cause of the changes in the bandgaps was investigated by performing DFT calculations for the La-containing systems NaLaTiO_4_, NaLaTiO_3_S, and NaLaTiO_2_S_2_ (Fig. 7c–e and S18), considering that these calculations for *f*-electron-containing systems are generally difficult. The calculated bandgap of NaLaTiO_3_S was narrower than that of NaLaTiO_4_ and closely approximated that of NaLaTiO_2_S_2_ (Table S9). The Ti-3d orbitals mainly contribute to the density of states (DOS) around the CBM in all cases (Fig. 7c–e). The DOS around the VBM of NaLaTiO_4_ was composed of O-2p orbitals, whereas those of NaLaTiO_3_S and NaLaTiO_2_S_2_ were mainly composed of S-3p orbitals in the S3 site adjacent to the La site, with additional contributions from the O-2p orbitals (Fig. S19). These results indicate that the narrow bandgap of the oxysulfide NaMTiO_2.2_S_1.8_ (M = Nd, Sm) is derived from the sizable contribution of the S-3p orbitals in the S3 site to the VBM.

### Photocatalytic activity

**Fig. 8:**
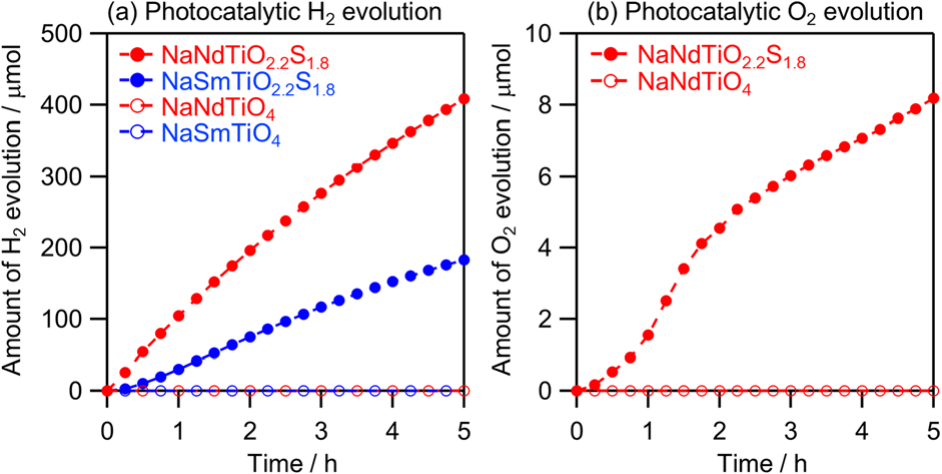
Time courses of photocatalytic (a) H_2_ and (b) O_2_ evolution on NaMTiO_4_ (M = Nd, Sm) and NaMTiO_2.2_S_1.8_ from water with an electron donor (S^2−^, SO_3_^2−^) and acceptor (Ag^+^), respectively, under visible light (400 < *λ* < 800 nm).

The photocatalytic H_2_ and O_2_ evolution activities of the new NaMTiO_2.2_S_1.8_ (M = Nd, Sm) and NaMTiO_4_ (H_2_S) (M = Pr, Eu, Gd) samples were evaluated under visible light irradiation in the presence of electron donors and acceptors, respectively (Fig. 8 and S20). All the compounds exhibit steady H_2_ evolution under visible light, with the activity of the Nd and Sm samples surpassing that of the others. This tendency is likely attributable to their higher phase purity. Their turnover numbers (TONs), defined as the number of moles of evolved H_2_ divided by the number of moles of NaMTiO_2.2_S_1.8_, were 2.5 (M = Nd) and 1.2 (M = Sm), indicating that H_2_ evolution proceeded photocatalytically. Notably, the oxide precursors NaMTiO_4_ (M = Nd, Sm) generated no H_2_ under visible light, indicating that bandgap narrowing *via* sulfurization rendered them responsive to visible light. The SEM images of NaMTiO_2.2_S_1.8_ (M = Nd, Sm) after H_2_ evolution revealed no changes (Fig. S21). The XRD patterns showed that, after the reaction, each of the two samples consisted of both the original NaMTiO_2.2_S_1.8_ and the corresponding hydrated compound (Fig. S22), indicating that interlayer hydration, along with partial H^+^ exchange, occurs during the reaction—an outcome similar to that when stirring in water in the dark. After the reaction, EDX of NaMTiO_2.2_S_1.8_ (M = Nd, Sm) revealed that the Na/Ti ratio significantly decreased from 1.12 to 0.67 (M = Nd) and 1.02 to 0.52 (M = Sm), accompanied by a moderate decrease in the S/Ti ratio from 1.73 to 1.32 (M = Nd) and 1.68 to 1.43 (M = Sm), respectively (Table S10). This decrease in sulfur content is also supported by X-ray photoelectron spectroscopy (XPS) analysis (Fig. S23). Furthermore, the decrease in the Na/Ti and S/Ti ratios was also evident after stirring in water in the dark (Na : Ti : S = 0.56 : 1 : 1.29 for M = Nd and 0.46 : 1 : 1.34 for M = Sm); thus, this compositional change proceeds chemically rather than photochemically. The above-mentioned DFT calculations and characterizations showed that the decrease in the Na/Ti and S/Ti ratios was due to proton exchange and S-to-O substitution at the S2(O2) site in NaMTiO_2.2_S_1.8_, respectively. Note that the S-to-O substitution at the S2(O2) site alters the shape of the absorption spectrum but not the absorption edge (Fig. S24). Therefore, we concluded that the NaMTiO_2.2_S_1.8_ (M = Nd, Sm) materials can function stably as photocatalysts, even after partial proton exchange and S-to-O substitution.

The evident O_2_ evolution activity of NaNdTiO_2.2_S_1.8_ (contrary to its oxide precursors) confirmed its water-oxidation capability under visible light irradiation (Fig. 8b). The O_2_ evolution was nonlinear, likely due to the photocatalytic oxidation of some sulfur species (*e.g.*, NaNdS_2_) present on the photocatalyst surface. Enabling the oxysulfide NaNdTiO_2.2_S_1.8_ to achieve overall visible-light-driven water splitting (*i.e.*, water splitting using a single photocatalyst) would require further optimization of the synthesis methods/conditions and appropriate surface modifications in the future. For example, enhancing crystallinity, controlling morphology, and tuning the exposed facets through flux synthesis may improve charge separation, thereby enhancing photocatalytic activity. Additionally, we expect interlayer modification (*e.g.*, loading a cocatalyst into the interlayer) of NaNdTiO_2.2_S_1.8_ to significantly improve its photocatalytic H_2_ and O_2_ evolution.

## Conclusions

In conclusion, we successfully synthesized a novel RP (*n* = 1) oxysulfide, NaMTiO_2.2_S_1.8_, with an “interlayer-active” nature through sulfurization of the layered oxide NaMTiO_4_ under H_2_S gas. The sulfur primarily occupies the apical oxygen sites, and the sulfur atoms adjacent to M predominantly contribute to the elevated VBM to enable NaMTiO_2.2_S_1.8_ to absorb visible light. This oxysulfide has both ion exchange and interlayer hydration capabilities, contrary to conventional RP oxysulfides such as M_2_Ti_2_O_5_S_2_, while also demonstrating photocatalytic hydrogen and oxygen evolution abilities under visible light irradiation. The discovery of an “interlayer-active” RP (*n* = 1) oxysulfide, NaMTiO_2.2_S_1.8_, enables new developments in various fields, including water splitting photocatalysis, *via* ion exchange reactions and interlayer hydration.

## Experimental section

### Materials

Na_2_CO_3_ (99.8%), La_2_O_3_ (99.99%), Nd_2_O_3_ (99.9%), Sm_2_O_3_ (99.9%), Eu_2_O_3_ (99.9%), Gd_2_O_3_ (99.9%), Er_2_O_3_ (99.9%), Y_2_O_3_ (99.99%), TiO_2_ (anatase form, 98.5%), 1 M HCl aqueous solution (aq), RhCl_3_·3H_2_O (95.0–102.0%), Na_2_S (98.0%), Na_2_SO_3_ (97.0%), AgNO_3_ (99.8%), and 1 M NaOH aqueous solution (aq.) were purchased from the FUJIFILM Wako Pure Chemical Corporation. Pr_2_O_3_ (99.9%) was purchased from Kojundo Chemical Laboratory Co., Ltd Na_3_IrCl_6_·*n*H_2_O (80.0%) was purchased from Kanto Chemical Co., Inc.

### Sample synthesis

NaMTiO_4_ samples (M = La, Pr, Nd, Sm, Eu, Gd, Er, and Y) were synthesized *via* solid-state reactions from a mixture of Na_2_CO_3_, M_2_O_3_, and TiO_2_ in a molar ratio of Na : M : Ti = 1.4 : 1 : 1, according to a method reported previously.^34^ The mixture was then calcinated in an alumina crucible at 1173–1323 K for 30 min in air (1173 K: M = La; 1273 K: M = Pr, Nd, Sm, Eu, Gd, Y; 1323 K: M = Er). NaMTiO_4−*x*_S_*x*_ (NaMTiO_4_ (H_2_S)) (*x* = 1.8; M = Nd, Sm) was synthesized by heating the obtained NaMTiO_4_ samples at 1223 K for 2 h under flowing H_2_S (flow rate: 50 (M = Sm) or 100 (other) mL min^−1^).

The ion-exchange behavior of NaMTiO_2.2_S_1.8_ was examined by stirring the compound for 24 h in an aqueous solution of HCl at room temperature (298 K) in the dark. The solution was prepared by adding 1 M aqueous HCl in distilled water in a molar ratio of H^+^ : Na^+^ = 1 : 1. To hydrate NaMTiO_2.2_S_1.8_ (M = Nd, Sm), the synthesized NaMTiO_2.2_S_1.8_ was stirred in the dark for 72 h at room temperature (298 K) or in hot (333 K) distilled water, after which the products were collected *via* filtration and dried naturally. After drying, the samples were heated in a vacuum oven at 303 K.

### Characterization

Powder XRD (PXRD; MiniFlex II, Rigaku, X-ray source: CuK_α_), UV-visible diffuse reflectance spectroscopy (Shimadzu, UV-2600i), scanning electron microscopy (SEM; NVision 40, Carl Zeiss-SIINT) and scanning transmission electron microscopy (STEM; JEOL, JEM-ARM200CF) equipped with EDX were used to characterize the samples. Synchrotron X-ray diffraction (SXRD; BL02B2, SPring-8, Japan, *λ* = 0.413922 Å) patterns were collected at room temperature. Indexing of the SXRD patterns and Rietveld refinement were conducted using JANA2006.^46^ The VESTA program was used to construct the crystal structures.^47^ The ionization energy was directly measured by PYS (BIP-KV201, Bunkoukeiki) in a vacuum (<5 × 10^−2^ Pa). Before the measurements, we calibrated the work function of a gold film (as a standard specimen), which corresponded to the Fermi energy from the vacuum level. X-ray photoelectron spectroscopy (XPS; JPS-9200, JEOL, X-ray source: Mg Kα) was employed for surface characterization. The XPS binding energies were calibrated using the 4f_7/2_ peak of Au (83.3 eV) deposited on the sample surface.

### Electrochemical measurement

Mott–Schottky plots were recorded in a three-electrode cell equipped with a Pt wire counter-electrode and Ag/AgCl reference electrode in a phosphate-buffered solution (0.1 M, pH = 12.0) using an electrochemical analyzer (VersaSTAT 4, Princeton Applied Research) with an amplitude of 10 mV and a frequency of 1000 Hz. The electrodes were prepared using the squeegee method. A particulate sample containing a small amount of water was coated onto a fluorine-doped tin oxide (FTO) conductive substrate and dried overnight at room temperature.

### DFT calculations

The electronic structures of NaLaTiO_4_, NaLaTiO_3_S, and NaLaTiO_2_S_2_ were calculated using the Cambridge Serial Total Energy Package (CASTEP).^48^ The calculated models of NaLaTiO_3_S and NaLaTiO_2_S_2_ assumed that O3 site and the O2 and O3 sites, respectively, were fully occupied by S (Fig. S18). The Perdew–Burke–Ernzerhof functional for solids (PBEsol) generalized gradient approximation (GGA) was used as the exchange–correlation functional,^49^ and the electronic states were expanded using a plane-wave basis set with a cut-off of 660 eV. The *k*-point meshes were set as 4 × 4 × 1. Before the PDOS calculation, geometry optimization was performed using the Broyden–Fletcher–Goldfarb–Shannon (BFGS) algorithm.

### Photocatalytic reactions

Photocatalytic reactions were carried out in a Pyrex glass reactor under an Ar flow (20 mL min^−1^). The quantity of evolved gases was determined using an online gas chromatograph (GC 3210D, GL Sciences, thermal conductivity detector, column packing: 5 Å molecular sieves, and Ar carrier gas). For the photocatalytic H_2_ evolution, the photocatalyst powders (0.05 g) were suspended in 180 mL of an aqueous Na_2_S/Na_2_SO_3_ solution (10 mM each), including RhCl_3_·3H_2_O as a precursor of the Rh cocatalyst (1 wt% as Rh metal). The photocatalysts were irradiated with visible light (*λ* > 400 nm) using a cutoff filter (HOYA; L42) from a 300 W Xe-arc lamp (PerkinElmer, Cermax PE300BF). For the photocatalytic O_2_ evolution, 0.05 g of IrO_2_-loaded photocatalysts was suspended in 180 mL of an aqueous AgNO_3_ solution (10 mM, pH = 8.6, adjusted using 1 M NaOH aq.) with La_2_O_3_ (0.05 g) as a buffer and irradiated with visible light (*λ* > 400 nm). IrO_2_ (1 wt%) was loaded onto the photocatalyst powder through adsorption in a colloidal IrO_2_ solution. IrO_2_ colloids were prepared as reported previously.^50^ The as-prepared powders were suspended in an aqueous solution containing the desired amount of IrO_2_ colloids and stirred for 40 min. This procedure allowed the IrO_2_ nanoparticles to be adsorbed onto the photocatalyst surface. The IrO_2_-loaded photocatalyst powder was collected *via* filtration and dried naturally. The TONs, defined as the number of moles of evolved H_2_ at 5 h (NaNdTiO_2.23_S_1.77_: 408 μmol, NaSmTiO_2.24_S_1.76_: 183 μmol) divided by the number of moles of photocatalyst (NaNdTiO_2.23_S_1.77_: 163 μmol, NaSmTiO_2.24_S_1.76_: 159 μmol), were calculated to be 2.5 (NaNdTiO_2.23_S_1.77_) and 1.2 (NaSmTiO_2.24_S_1.76_), respectively.

## Author contributions

Y. I. designed the study, with advice from H. S., D. K. and R. A. Rietveld refinements were initially performed by D. K. and then finalized by Y. I. All remaining experiments were performed by Y. I. All authors discussed the results; Y. I. wrote the manuscript and H. S. edited it, with discussions mainly with Y. I., H. S., D. K. and R. A.

## Conflicts of interest

There are no conflicts to declare.

## Supplementary Material

SC-016-D5SC04851F-s001

## Data Availability

All data are available in the main manuscript and the SI. XRD patterns, EDX, SEM images, crystal structures, structural analysis from Rietveld refinement, lattice parameters obtained *via* Le Bail analysis, HAADF-STEM images, STEM-EDX, calculated models and DOS&PDOS, PYS, UV-vis diffuse reflectance spectra and photocatalytic activities. See DOI: https://doi.org/10.1039/d5sc04851f.
