# Supplementary material for: Interlayer-active layered oxysulfides NaMTiO2.2S1.8 (M = Nd, Sm) with an n = 1 Ruddlesden–Popper structure acting as photocatalysts for visible light water splitting
Source: Chem Sci. 2025 Aug 13;16(36):16534–41. doi: 10.1039/d5sc04851f (PMC12365923; doi:10.1039/d5sc04851f)
Supplement: SC-016-D5SC04851F-s001 [file SC-016-D5SC04851F-s001.pdf]

Supplementary Information (SI) for Chemical Science.
This journal is © The Royal Society of Chemistry 2025

Supporting Information

Interlayer-active layered oxysulfides $NaMTiO_{2.2}S_{1.8}$ (M = Nd, Sm) with an *n* = 1 Ruddlesden–Popper structure acting as photocatalysts for visible light water splitting

Yusuke Ishii,[a] Hajime Suzuki,*[a] Daichi Kato,[a] Osamu Tomita,[a] Akinobu Nakada,[a] Ryu Abe*[a]

[a] Department of Energy and Hydrocarbon Chemistry, Graduate School of Engineering, Kyoto University, Katsura, Nishikyo-ku, Kyoto 615-8510, Japan

* Corresponding Author: suzuki.hajime.7x@kyoto-u.ac.jp (H. S.), ryu-abe@scl.kyoto-u.ac.jp (R. A.).

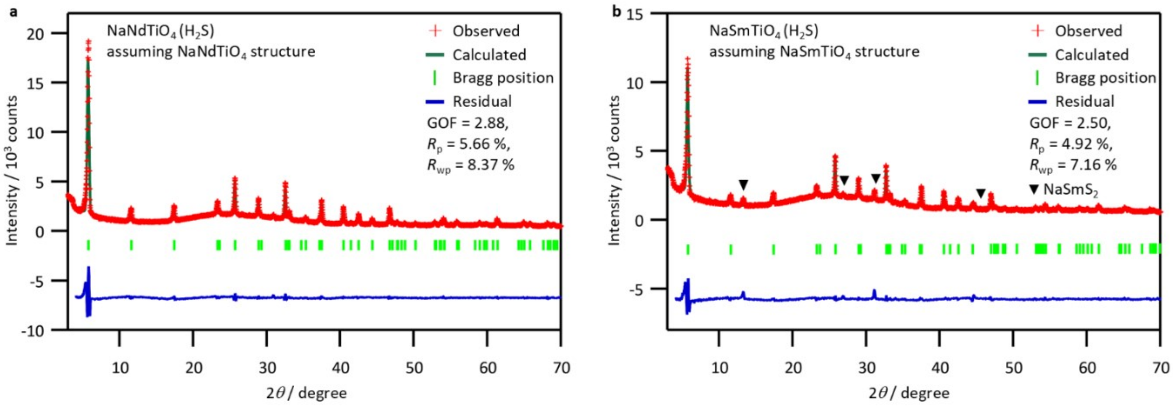

Figure S1. Le Bail refinement of the XRD patterns of (a) $NaNdTiO_4$ (H_2S) and (b) $NaSmTiO_4$ (H_2S) performed using Cu $K\alpha_1$ (λ = 1.54056 Å) and $K\alpha_2$ (λ = 1.54440 Å) radiation, assuming $NaMTiO_4$ (M = Nd, Sm) structures, respectively. Triangles denote the peaks of the byproduct $NaSmS_2$ (ICSD #644974).

Table S1. Na/Ti, M/Ti (M = Nd, Sm), and S/Ti atomic ratios, determined by EDX measurement, of $NaMTiO_4$ (H_2S).

$NaMTiO_4$ (H_2S)	Na/Ti	M/Ti	S/Ti
M = Nd	1.12	1.08	1.73
M = Sm	1.06	0.95	1.68

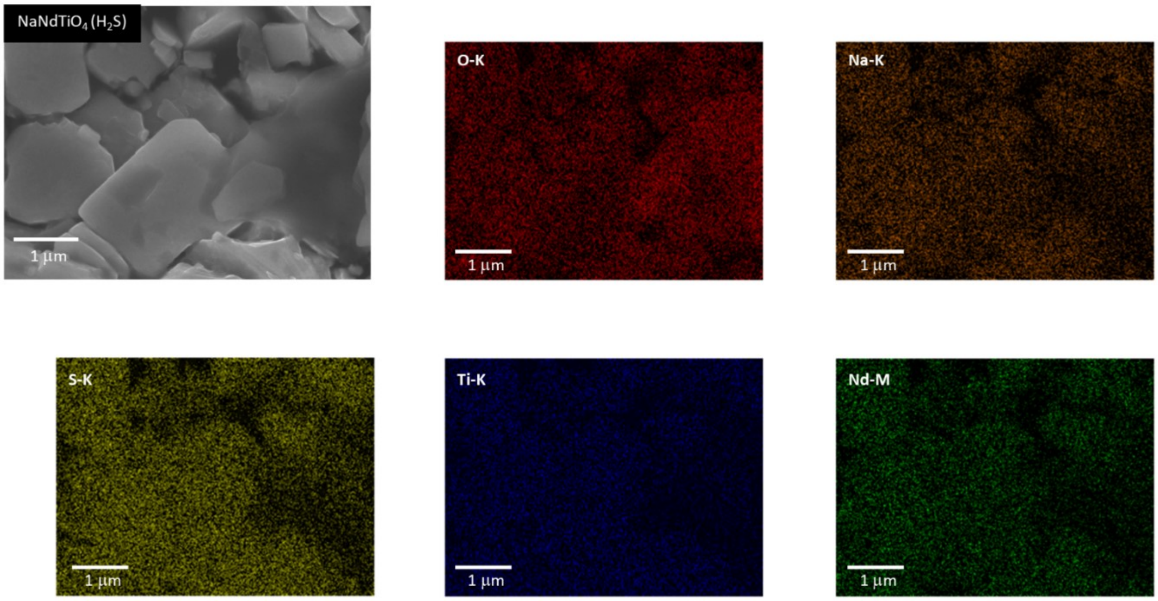

Figure S2. SEM images and SEM-EDX elemental mappings of $NaNdTiO_4$ (H_2S).

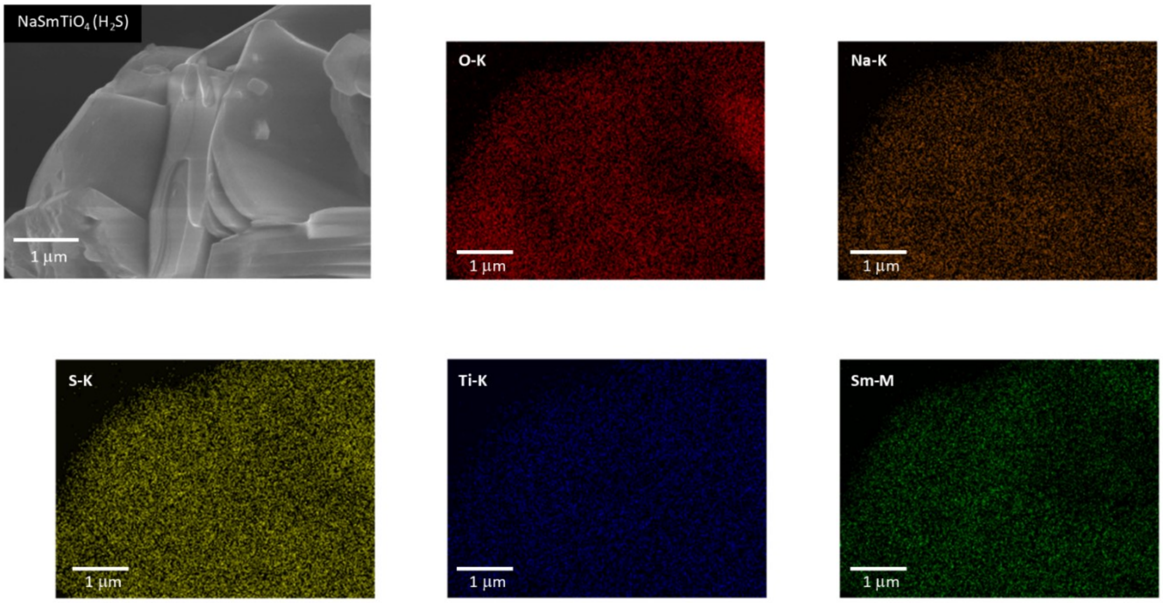

Figure S3. SEM images and SEM-EDX elemental mappings of $NaSmTiO_4$ (H_2S).

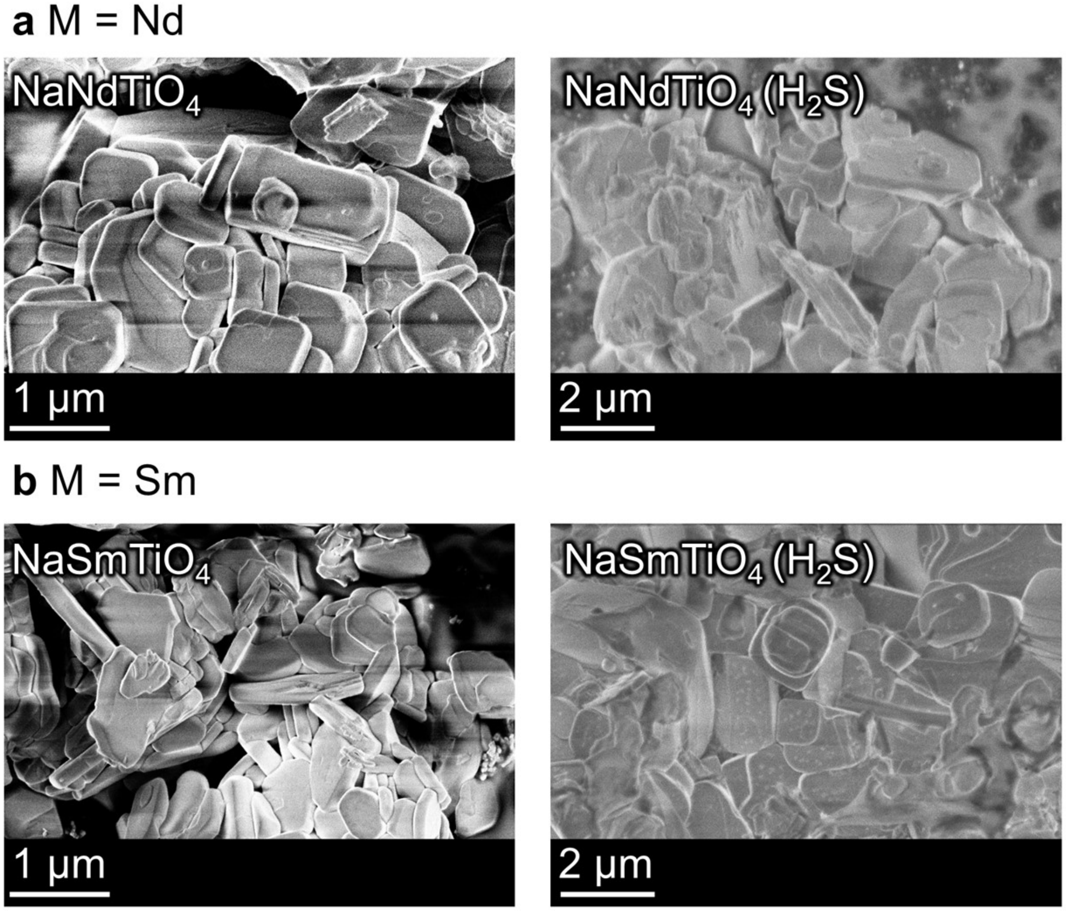

Figure S4. SEM images of $NaMTiO_4$ (M = (a) Nd, (b) Sm) and the sulfurized product of $NaMTiO_4$ ($NaMTiO_4$ (H_2S)).

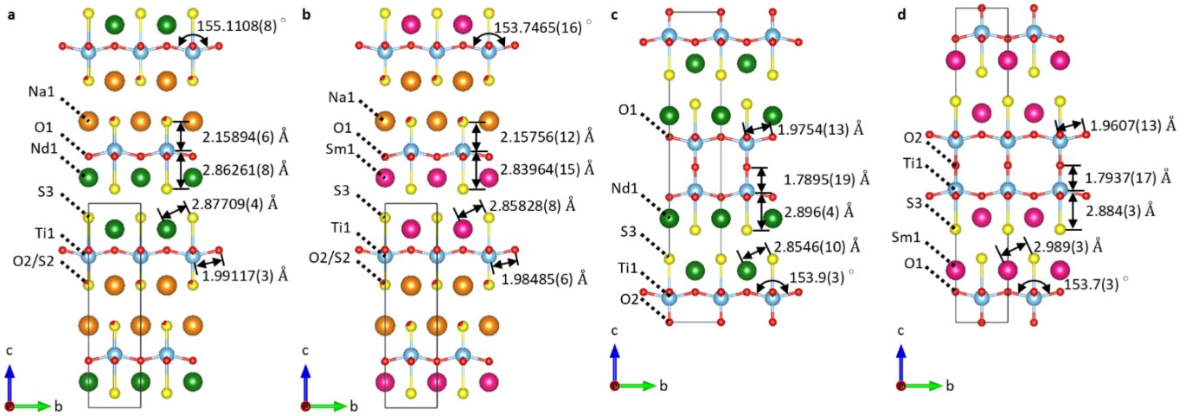

Figure S5. Crystal structures of (a) $NaNdTiO_{2.23}S_{1.77}$, (b) $NaSmTiO_{2.24}S_{1.76}$, (c) $Nd_2Ti_2O_5S_2$, and (d) $Sm_2Ti_2O_5S_2$.

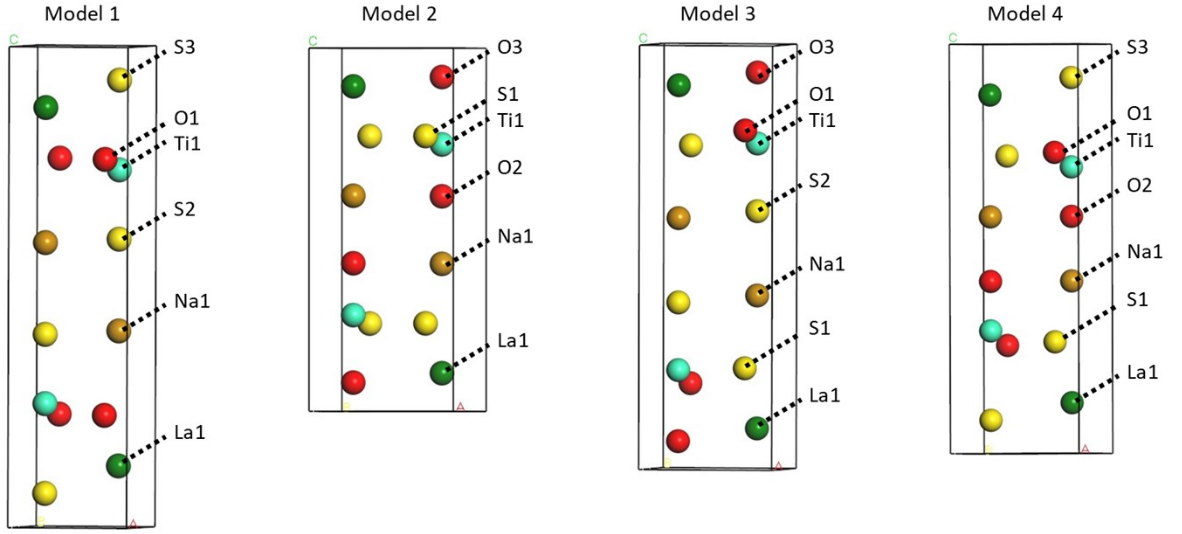

Figure S6. Anion-ordered structural models of $NaLaTiO_2S_2$ (space group: *P*1) were used for DFT calculations (Models 1–4).

Table S2. Energy differences (in eV) of Models 2–4 relative to the most stable Model 1, as shown for each structural model in Figure S6.

	Model2	Model3	Model4
Energy difference / eV	5.33	4.48	2.11

Table S3. Ratios of the calculated lattice parameters (*b*/*a*, *c*/*a*, and *c*/*b*) of $NaLaTiO_2S_2$ using structural Models 1–4 (shown in Figure S6) along with the experimental lattice parameters (*b*/*a*, *c*/*a*, and *c*/*b*) of $NaNdTiO_{2.23}S_{1.77}$ and $NaSmTiO_{2.24}S_{1.76}$.

Parameters	Model 1	Model 2	Model 3	Model 4	$NaNdTiO_{2.23}S_{1.77}$	$NaSmTiO_{2.24}S_{1.76}$
b/*a*	1.00003	1.00000	0.832095	0.814116	1	1
c/*a*	3.92521	2.44982	3.12135	3.03249	3.91944	3.93918
c/*b*	3.92510	2.44981	3.75119	3.72489	3.91944	3.93918

Density functional theory (DFT) calculations (Figure S6) were performed to gain insight into the site selectivity of sulfur in $NaMTiO_{2.2}S_{1.8}$. As DFT calculations for f-electron-containing systems are generally difficult, La-substituted models (Models 1–4) of $NaLaTiO_2S_2$ were employed. The stability and lattice parameters (*b*/*a*, *c*/*a*, and *c*/*b*) of various anion-ordered models (Models 1–4) of $NaLaTiO_2S_2$ were compared. In Model 1, the O2 and O3 sites are fully occupied by sulfur. This model is the most stable, and its lattice parameters are the closest to those of $NaMTiO_{2.2}S_{1.8}$ obtained from Rietveld refinement (Tables S2 and S3).

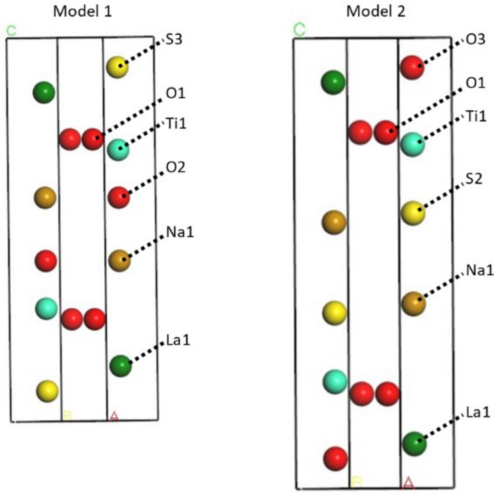

Figure S7. Anion-ordered structural models of $NaLaTiO_3S$ used for DFT calculations (Models 1 and 2). The preferential substitution of sulfur at the O3 site compared to the O2 site in $NaMTiO_{2.2}S_{1.8}$ is also supported by DFT calculations using various $NaLaTiO_3S$ models (Figure S7), which indicated that the O-to-S substitution at the O3 site was 1.02 eV more stable than at the O2 site.

Table S4. Refined atomic parameters of $NaNdTiO_{2.23}S_{1.77}$ from the SXRD data of $NaNdTiO_4$ (H_2S) (Figure 3a) using the structural model of $NaNdTiO_{2.23}S_{1.77}$ shown in Figure S5a.

Site	Occupancy	x	y	z	U_{iso} (Å)
Nd1	1	0	0.5	0.87386(11)	0.0045(4)
Na1	1	0	0.5	0.5980(7)	0.040(4)
Ti1	1	0	0.5	0.2584(3)	0.0058(10)
O1	1	0	0	0.2303(7)	0.003(2)
S2	0.77(3)	0	0.5	0.4001(5)	0.015(3)
O2	0.23(3)	0	0.5	0.4001(5)	0.015(3)
S3	1	0	0.5	0.0706(4)	0.0041(11)

$P4/nmm$, $a = b$ = 3.88877(5) Å, c = 15.2418(3) Å, R_{wp} = 7.81 %, R_p = 5.71 %, GOF = 1.39

Table S5. Refined atomic parameters of $NaSmTiO_{2.24}S_{1.76}$ from the SXRD data of $NaSmTiO_4$ (H_2S) (Figure 3b) using the structural model of $NaSmTiO_{2.24}S_{1.76}$ shown in Figure S5b.

Site	Occupancy	x	y	z	U_{iso} (Å)
Sm1	1	0	0.5	0.8749(2)	0.0045(7)
Na1	1	0	0.5	0.5980(14)	0.051(8)
Ti1	1	0	0.5	0.2567(6)	0.0058(19)
O1	1	0	0	0.2271(14)	0.01[a]
S2	0.76(6)	0	0.5	0.3984(9)	0.013(5)
O2	0.24(6)	0	0.5	0.3984(9)	0.013(5)
S3	1	0	0.5	0.0702(8)	0.004(2)

[a]Fixed during refinement

$P4/nmm$, $a = b$ = 3.86598(11) Å, c = 15.2288(8) Å, R_{wp} = 9.75 %, R_p = 6.82 %, GOF = 2.00

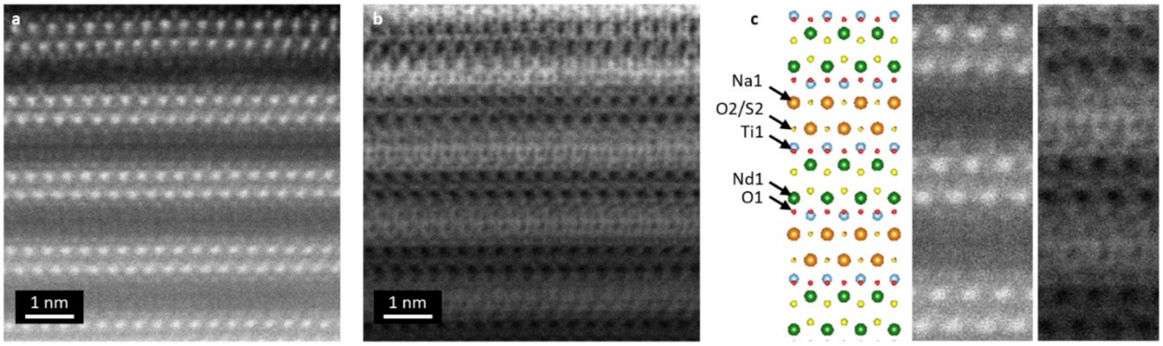

Figure S8. STEM images of $NaNdTiO_{2.2}S_{1.8}$ in the [100] direction. (a) HADDF, (b) ABF, and (c) their enlargements with the crystal structure of $NaNdTiO_{2.2}S_{1.8}$.

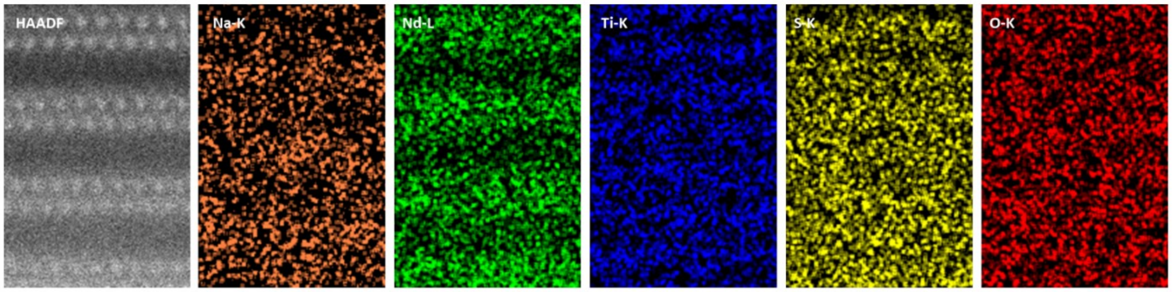

Figure S9. HAADF-STEM images and elemental mappings of $NaNdTiO_{2.2}S_{1.8}$ in the [100] direction.

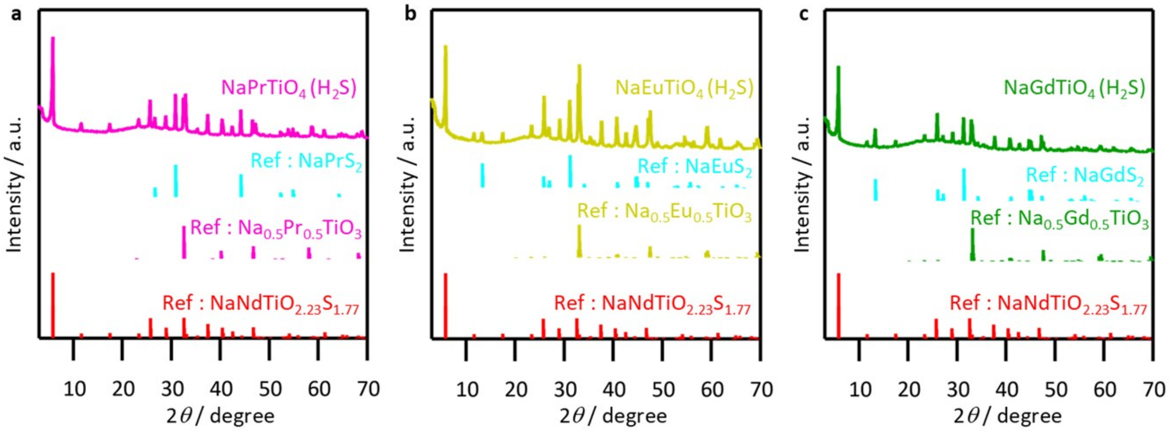

Figure S10. XRD patterns of the sulfurized product of $NaMTiO_4$ ($NaMTiO_4$ (H_2S)) (M = (a) Pr, (b) Eu, (c) Gd), along with reference patterns of $Na_{0.5}Pr_{0.5}TiO_3$ (ICSD #167784), $NaPrS_2$ (ICSD #644940), $Na_{0.5}Eu_{0.5}TiO_3$ (ICSD #245521), $NaEuS_2$ (ICSD #199758), $Na_{0.5}Gd_{0.5}TiO_3$ (ICSD #245627), $NaGdS_2$ (ICSD #48243), and $NaNdTiO_{2.23}S_{1.77}$ (this work).

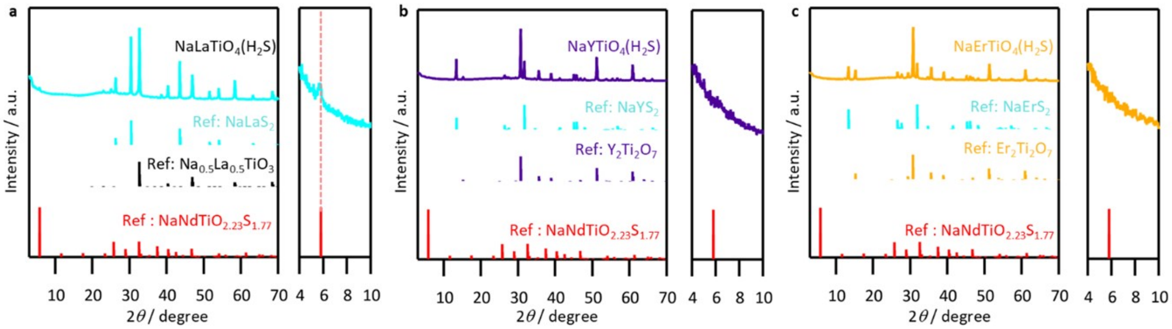

Figure S11. XRD patterns of the sulfurized product of $NaMTiO_4$ ($NaMTiO_4$ (H_2S)) (M = (a) La, (b) Y, (c) Er), along with the reference patterns of $NaLaS_2$ (ICSD #192109), $Na_{0.5}La_{0.5}TiO_3$ (ICSD #51029), $NaYS_2$ (ICSD #195944), $Y_2Ti_2O_7$ (ICSD #66874), $NaErS_2$ (ICSD #73481), $Er_2Ti_2O_7$ (ICSD #24209), and $NaNdTiO_{2.23}S_{1.77}$ (this work).

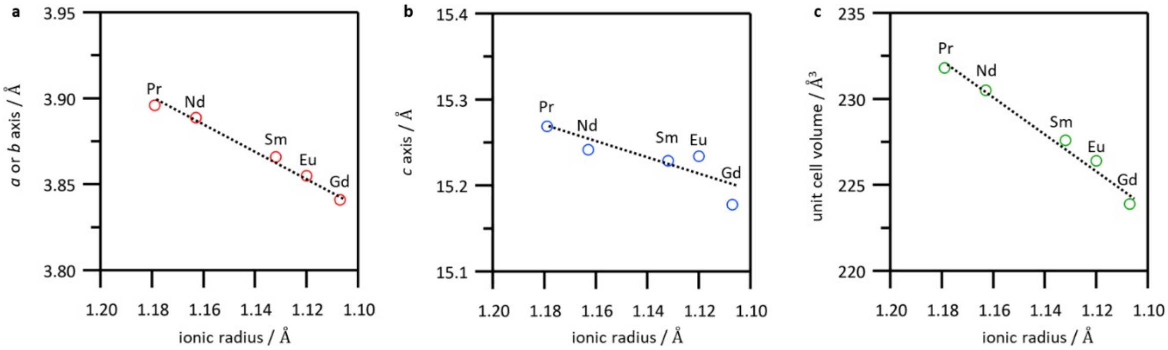

Figure. S12. Variations in the lattice parameters along the (a) *a*- or *b*-axis and (b) *c*-axis and (c) unit cell volume of $NaMTiO_{4-x}S_x$ (M = Pr, Eu, and Gd) obtained *via* Le Bail analysis using the $NaNdTiO_{2.23}S_{1.77}$ model and $NaMTiO_{2.2}S_{1.8}$ (M = Nd, Sm) obtained *via* Rietveld refinements as a function of the ionic radius of the lanthanide ions.

Table S6. Calculated energy barrier[1] for the removal of Na from the lattice of $NaMTiO_4$ (M = La, Y).

	M = La	M = Y
$Na_3M_4Ti_4O_{16}$	–21626.96	–21648.61
Na	–1304.94	–1304.94
$Na_4M_4Ti_4O_{16}$	–22937.27	–22959.29
Ea	5.37	5.74

$Ea = E_{Na\text{-}defect} + E_{Na} – E_{perfect}$

For M = La, the intensity of the peak corresponding to the $NaMTiO_{4-x}S_x$ was much lower than that for M = Pr–Gd. This is likely due to the energy required to remove Na from the precursor oxide $NaMTiO_4$, which is related to the ionic radius of M. The removal energy[1] was the lowest for the oxide with the largest La radius, leading to its structural collapse during sulfurization and thus hindering the formation of the oxysulfide. (Table S6).

For M = Y, Er, the main phase obtained by the sulfurization of $NaMTiO_4$ was the pyrochlore structure $M_2Ti_2O_7$ (M = Y, Er), which differed from the main impurity phase of the perovskite structure $Na_{0.5}M_{0.5}TiO_3$ obtained by La–Gd. $M_2Ti_2O_7$ may be more stable than $Na_{0.5}M_{0.5}TiO_3$ and the oxysulfide $NaMTiO_{4-x}S_x$, leading to its preferential formation over the oxysulfide.

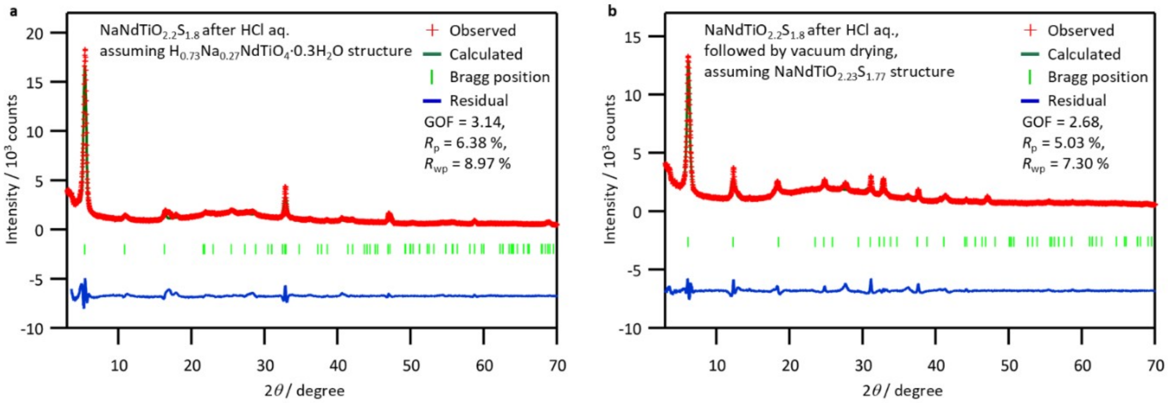

Figure S13. Le Bail refinement of the XRD pattern of $NaNdTiO_{2.2}S_{1.8}$ after (a) stirring in an HCl aqueous solution for 24 h, (b) followed by vacuum drying, performed using Cu $K\alpha_1$ (λ = 1.54056 Å) and $K\alpha_2$ (λ = 1.54440 Å) radiation, assuming (a) $H_{0.73}Na_{0.27}NdTiO_4{\cdot}0.3H_2O$ and (b) $NaNdTiO_{2.23}S_{1.77}$ structures, respectively.

Table S7. Lattice parameters along the *a*- or *b*-axis and *c*-axis of $NaMTiO_{2.2}S_{1.8}$ (M = (a) Nd and (b) Sm) obtained *via* Le Bail analysis using the $H_{0.73}Na_{0.27}NdTiO_4{\cdot}0.3H_2O$ and $NaMTiO_{2.2}S_{1.8}$ models before and after stirring in an HCl aqueous solution for 24 h or hot distilled water (HW) for 72 h, followed by vacuum drying. The representative results obtained by the analysis are shown in Figure S13.

a $NaNdTiO_{2.2}S_{1.8}$

$NaNdTiO_{2.2}S_{1.8}$	before	after HCl	after HCl & vacuum dry	after HW	after HW & vacuum dry
a(*b*) / Å	3.88875(5)	4.142(8)	3.864(3)	4.145(6)	3.897(4)
c / Å	15.2418(3)	16.31(4)[1)]	14.42(3)	16.22(3)[2)]	14.50(3)

1) True value: 32.62(7) (=2×c). 2) True value: 32.44(5) (=2×c).

b $NaSmTiO_{2.2}S_{1.8}$

$NaSmTiO_{2.2}S_{1.8}$	before	after HCl	after HCl & vacuum dry	after HW	after HW & vacuum dry
a(*b*) / Å	3.86598(11)	4.136(9)	3.837(2)	4.080(6)	3.828(2)
c / Å	15.2288(8)	16.26(3)[1)]	14.51(2)	16.20(4)[2)]	14.60(2)

1) True value: 32.51(5) (=2×c). 2) True value: 32.39(8) (=2×c).

Table S8. Na/Ti atomic ratios, determined by EDX measurement, of $NaMTiO_{2.2}S_{1.8}$ (M = Nd, Sm) before and after stirring under dark conditions in an HCl aqueous solution for 24 h or hot distilled water for 72 h.

	Before	After HCl aq.	After water
$NaNdTiO_{2.2}S_{1.8}$	1.12	0.18	0.27
$NaSmTiO_{2.2}S_{1.8}$	1.06	0.11	0.18

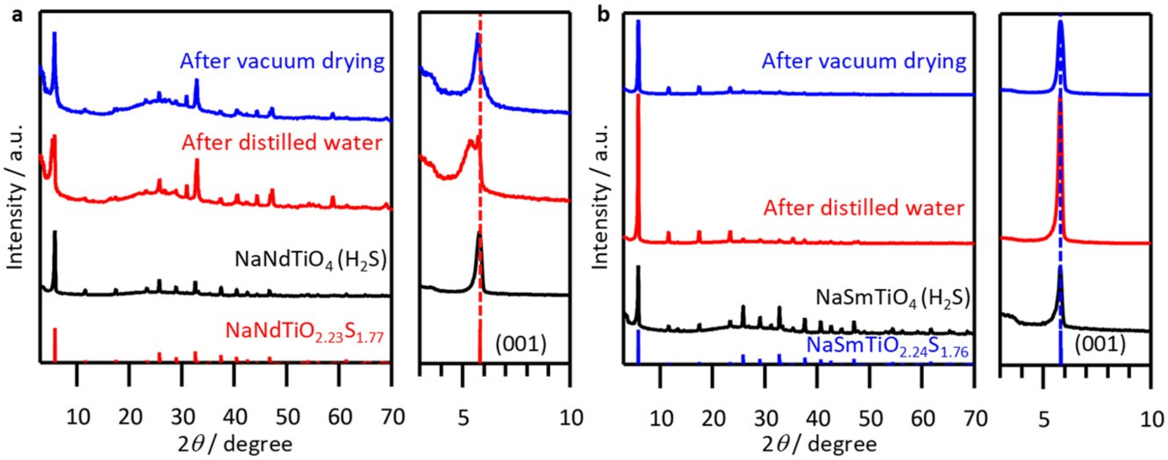

Figure S14. XRD patterns of the sulfurized products, $NaMTiO_{2.2}S_{1.8}$ (M = (a) Nd, (b) Sm) and the samples after stirring under dark conditions in distilled water for 72 h, followed by vacuum drying, along with the reference patterns of $NaNdTiO_{2.23}S_{1.77}$ (this work) and $NaSmTiO_{2.24}S_{1.76}$ (this work).

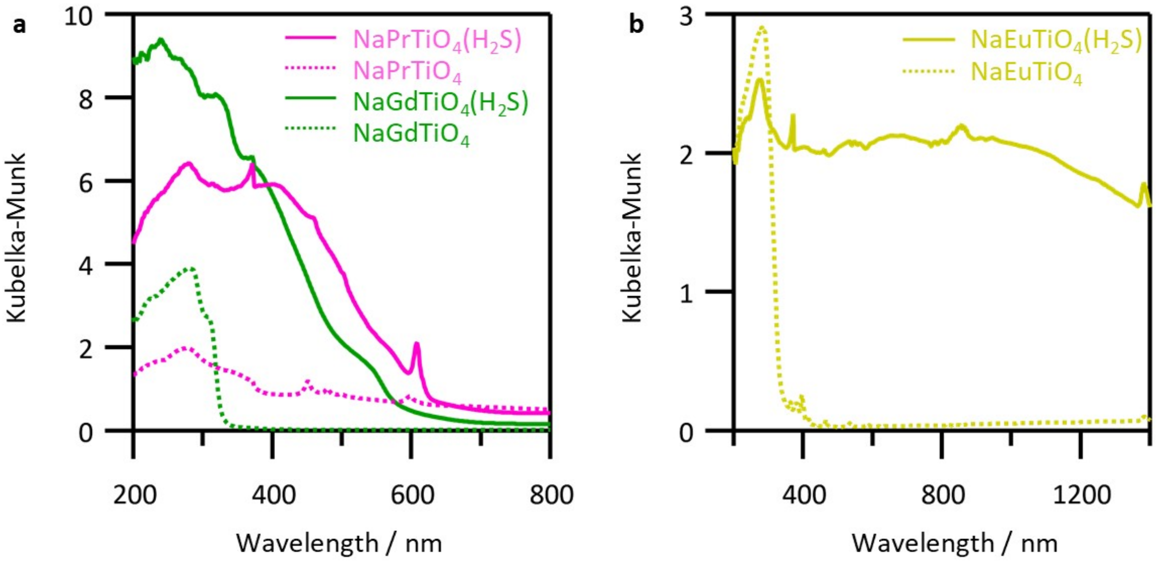

Figure S15. UV−vis diffuse reflectance spectra of (a) $NaMTiO_4$ and $NaMTiO_4$ (H_2S) (M = Pr, Gd) and (b) $NaEuTiO_4$ and $NaEuTiO_4$ (H_2S).

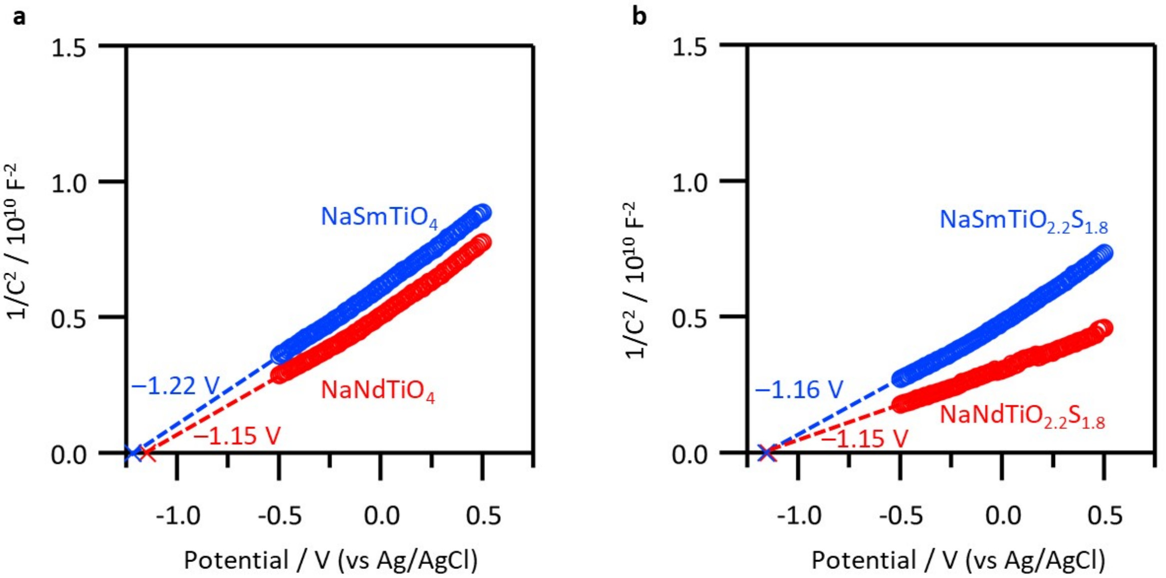

Figure S16. Mott-Schottky plots for (a) $NaMTiO_4$ and (b) $NaMTiO_{2.2}S_{1.8}$ (M = Nd, Sm).

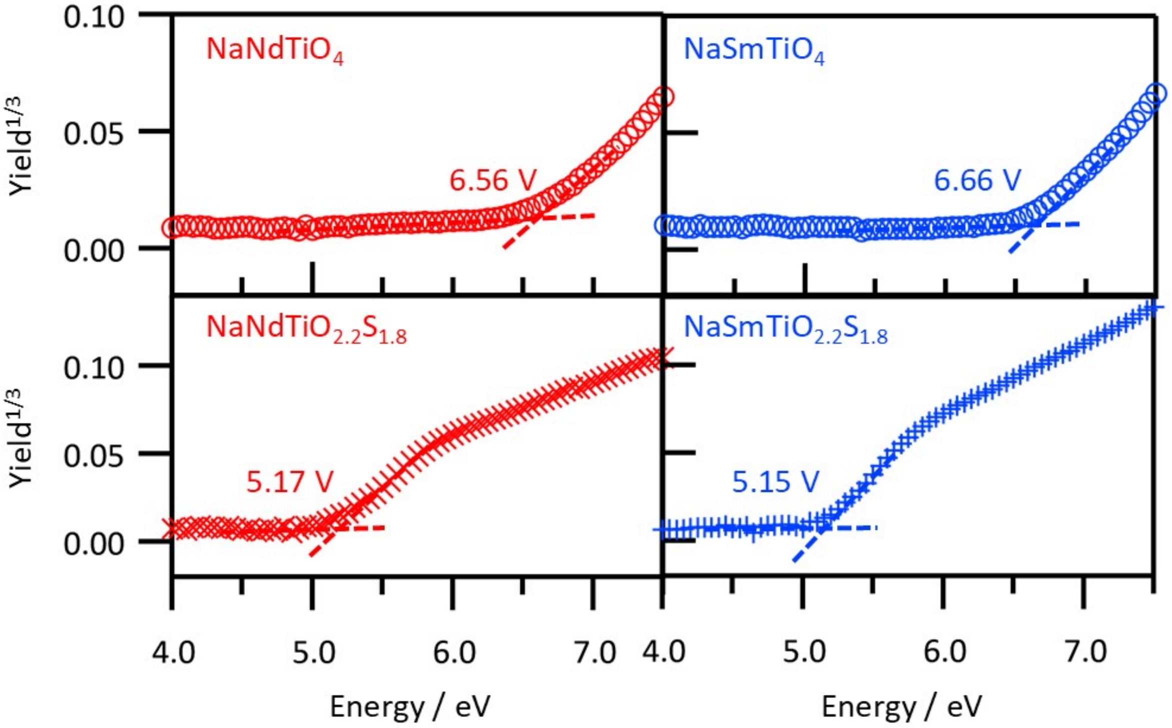

Figure S17. Photoelectron yield spectroscopy (PYS) spectra of $NaMTiO_4$ and $NaMTiO_{2.2}S_{1.8}$ (M = Nd, Sm).

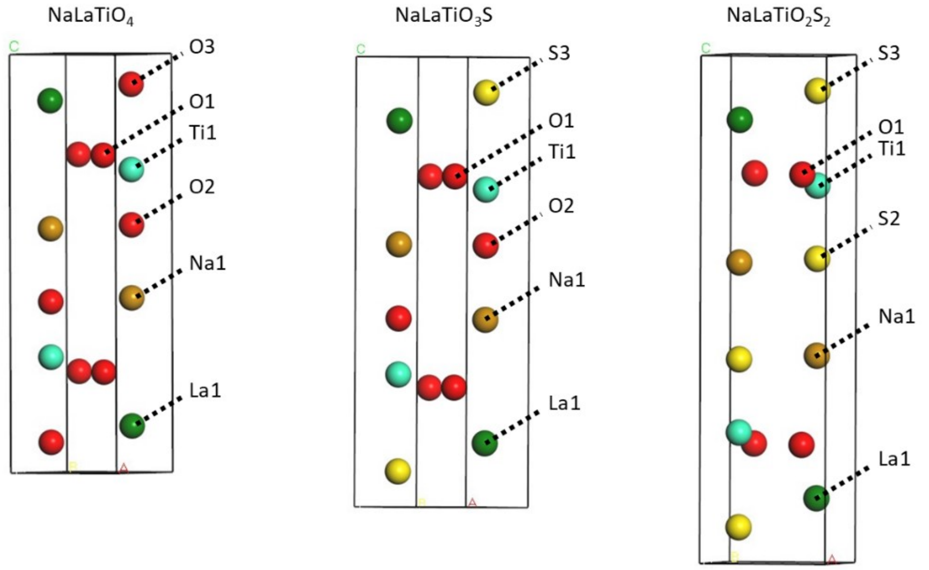

Figure S18. Structural models of $NaLaTiO_4$, $NaLaTiO_3S$, and $NaLaTiO_2S_2$ used for DFT calculations.

Table S9. Bandgaps of $NaLaTiO_4$, $NaLaTiO_3S$, and $NaLaTiO_2S_2$, calculated by DFT calculations.

	$NaLaTiO_4$	$NaLaTiO_3S$	$NaLaTiO_2S_2$
Bandgap / eV	2.57	1.22	1.15

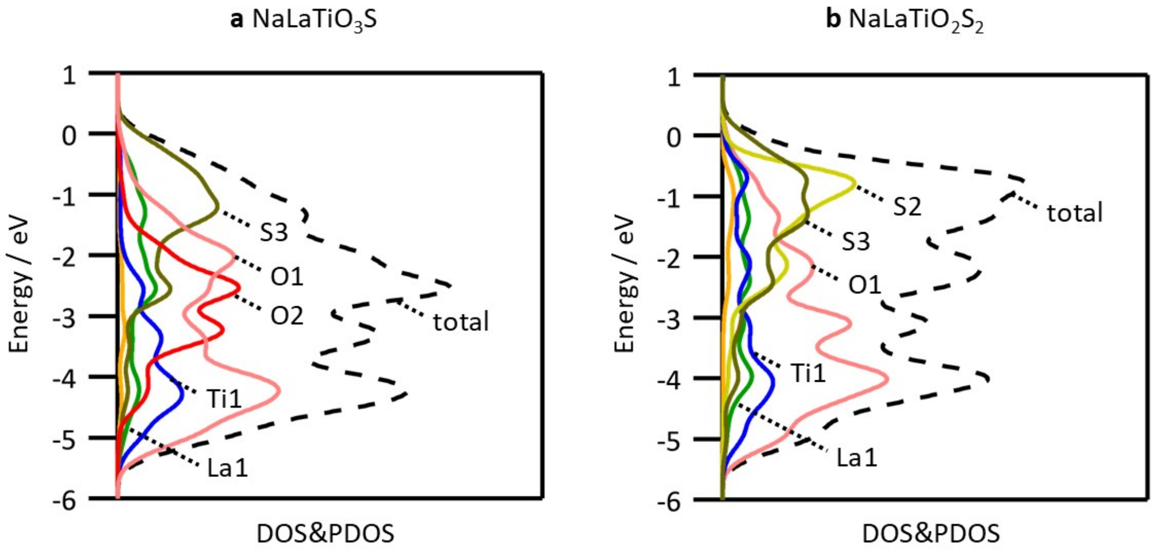

Figure S19. Enlarged DOS and PDOS near the VBM of (a) $NaLaTiO_3S$ and (b) $NaLaTiO_2S_2$ calculated using the structural models shown in Figure S18.

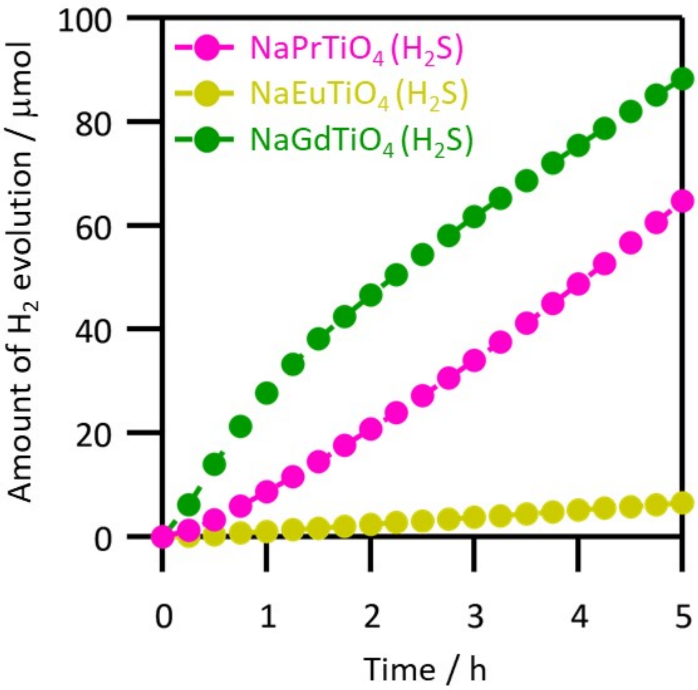

Figure S20. Time courses of photocatalytic H_2 evolution on $NaMTiO_4$ (H_2S) (M = Pr, Eu, Gd) from water with an electron donor (S^{2-}, SO_3^{2-}) under visible light ($400 < \lambda < 800$ nm).

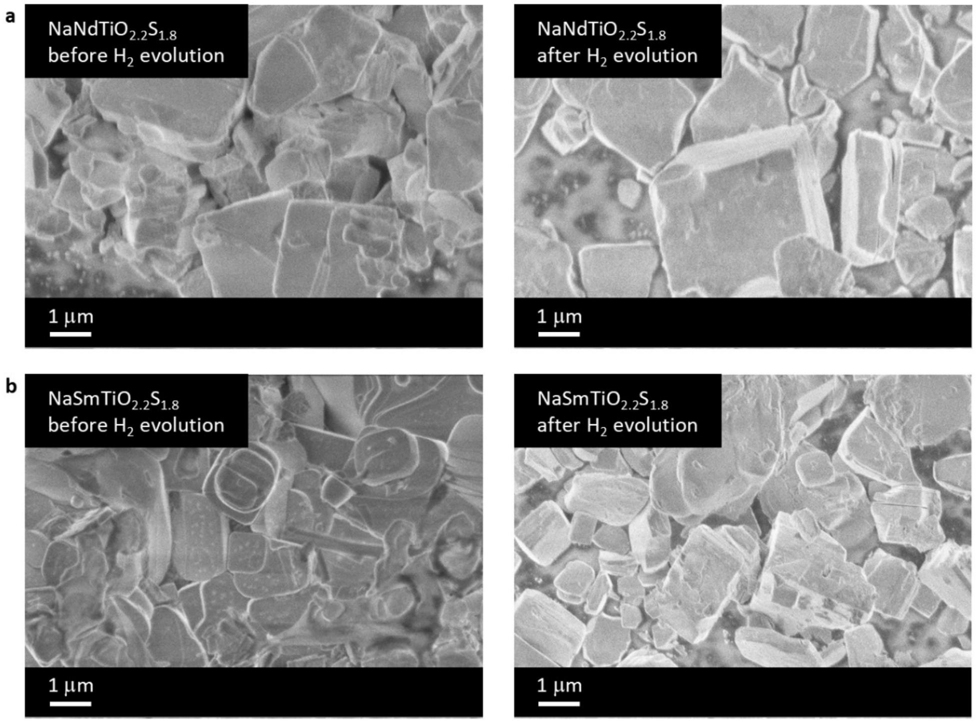

Figure S21. SEM images of (a) $NaNdTiO_{2.2}S_{1.8}$ and (b) $NaSmTiO_{2.2}S_{1.8}$ before and after the photocatalytic H_2 evolution reaction.

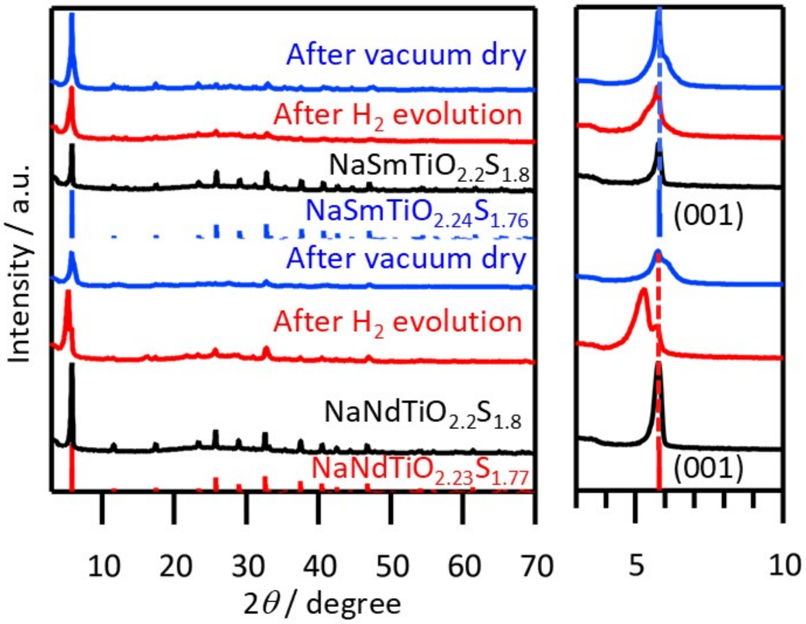

Figure S22. XRD patterns of $NaNdTiO_{2.2}S_{1.8}$, $NaSmTiO_{2.2}S_{1.8}$ and the samples after the photocatalytic H_2 evolution reaction, followed by vacuum drying, along with reference patterns of $NaNdTiO_{2.23}S_{1.77}$ (this work) and $NaSmTiO_{2.24}S_{1.76}$ (this work).

Table S10. Na/Ti, M/Ti (M = Nd, Sm), and S/Ti atomic ratios, determined by EDX measurement, of $NaMTiO_{2.2}S_{1.8}$ (M = (a) Nd, (b) Sm) before and after photocatalytic H_2 evolution in a Na_2S/Na_2SO_3 aqueous solution under visible light or stirring in distilled water for 72 h under dark conditions.
The Na/Ti and S/Ti ratios of $NaMTiO_{2.2}S_{1.8}$ (M = Nd, Sm) after stirring in water in the dark were lower than those after photocatalytic H_2 evolution, likely due to the longer reaction time (72 h for stirring vs. 5 h for photocatalysis).

a $NaNdTiO_{2.2}S_{1.8}$

$NaNdTiO_{2.2}S_{1.8}$	Na/Ti	Nd/Ti	S/Ti
Before	1.12	1.08	1.73
After H_2 evolution	0.67	0.98	1.32
After water	0.56	1.17	1.29

b $NaSmTiO_{2.2}S_{1.8}$

$NaSmTiO_{2.2}S_{1.8}$	Na/Ti	Nd/Ti	S/Ti
Before	1.06	0.95	1.68
After H_2 evolution	0.52	1.29	1.43
After water	0.46	1.10	1.34

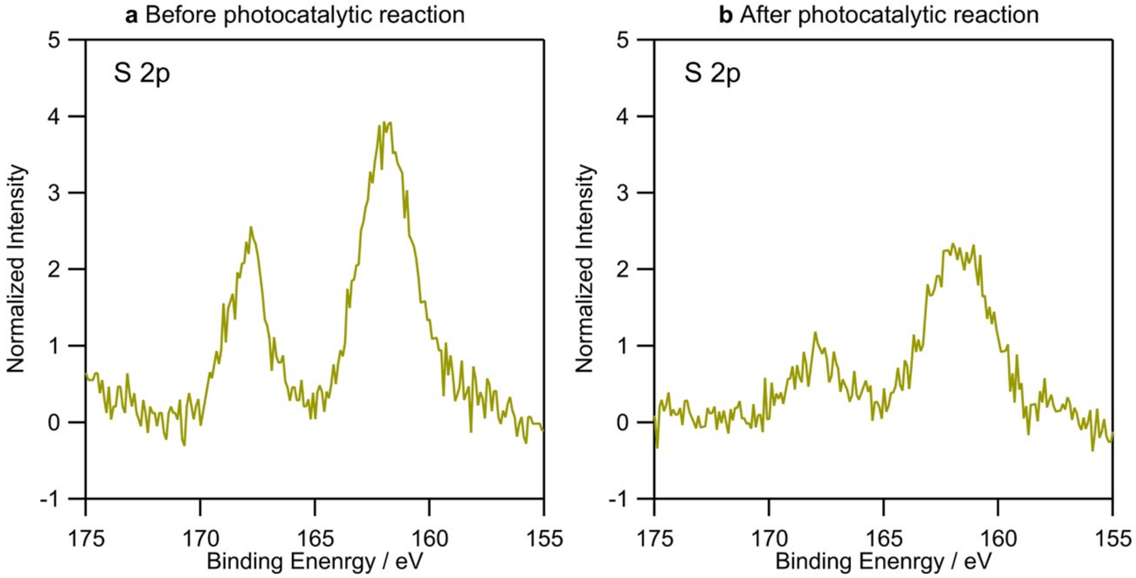

Figure S23. S 2p XPS profiles of $NaNdTiO_{2.2}S_{1.8}$ (a) before and (b) after the photocatalytic H_2 evolution reaction. Each intensity is normalized to the Nd $4d_{5/2}$ peak.

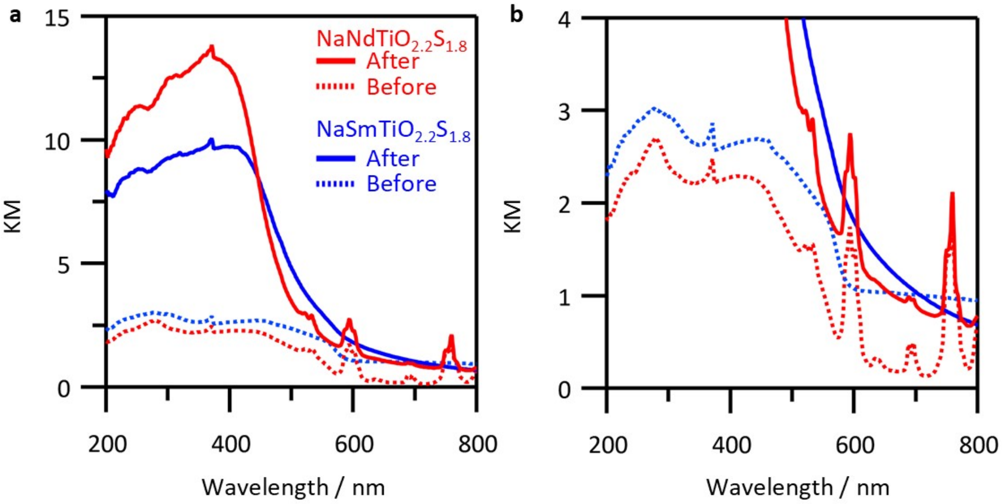

Figure S24. UV−vis diffuse reflectance spectra of $NaNdTiO_{2.2}S_{1.8}$ (M = Nd, Sm) before and after the photocatalytic H_2 evolution reaction, followed by vacuum drying.

References

1 Y. Bao, S. Du, Y. Qi, G. Li, P. Zhang, G. Shao and F. Zhang, *Adv. Mater.*, 2021, **33**, e2101883.
